# Filtered Cerebrospinal Fluid From Patients With Amyotrophic Lateral Sclerosis Displays an Altered Proteome and Affects Motor Phenotype in a Mouse Model

**DOI:** 10.7759/cureus.32980

**Published:** 2022-12-26

**Authors:** Vishal Venkatraman, Anthony J Filiano, Li Xu, Leonard Collins, Emily Luo, Katelyn M Ripple, George C de Castro, Jane-Valeriane K Boua, Choiselle Marius, Charles Giamberardino, Shivanand P Lad, Taufika Islam Williams, Michael S Bereman, Richard S Bedlack

**Affiliations:** 1 Department of Neurosurgery, Duke University Medical Center, Durham, USA; 2 Molecular Education, Technology and Research Innovation Center (METRIC), North Carolina State University, Raleigh, USA; 3 Department of Biological Sciences, North Carolina State University, Raleigh, USA; 4 Department of Neurology, Duke University Medical Center, Durham, USA

**Keywords:** mouse model, csf proteomics, csf filtration, neurapheresis, amyotrophic lateral sclerosis

## Abstract

Introduction: Cerebrospinal fluid (CSF) has been implicated in amyotrophic lateral sclerosis (ALS) due to its ability to spread inflammatory proteins throughout the nervous system. We hypothesized that filtration of the CSF could remove pathogenic proteins and prevent them from altering motor phenotypes in a mouse model.

Methods: We filtered the CSF from 11 ALS patients via 100 kilodaltons (kD) molecular weight cut-off filters. We used mass spectrometry-based discovery proteomics workflows to compare protein abundances before and after filtration. To test the effects of CSF filtration on motor function, we injected groups of mice with saline, filtered ALS-CSF, or unfiltered ALS-CSF (n=12 per group) and assessed motor function via pole descent and open field tests.

Results: We identified proteins implicated in ALS pathogenesis and showed that these were removed in significant amounts in our workflow. Key filtered proteins included complement proteins, chitinases, serine protease inhibitors, and neuro-inflammatory proteins such as amyloid precursor protein, chromogranin A, and glial fibrillary acidic protein. Compared to the filtered ALS-CSF mice, unfiltered ALS-CSF mice took longer to descend a pole (10 days post-injection, 11.14 seconds vs 14.25 seconds, p = 0.02) and explored less on an open field (one day post-injection, 21.81 m vs 16.83 m, p = 0.0004).

Conclusions:We demonstrated the ability to filter proteins from the CSF of ALS patients and identified potentially pathologic proteins that were reduced in quantity. Additionally, we demonstrated the ability of unfiltered ALS-CSF to induce motor deficits in mice on the pole descent and open field tests and showed that filtration could prevent this deficit. Given the lack of effective treatments for ALS, this could be a novel solution for patients suffering from this deadly and irreversible condition.

## Introduction

Studies have explored the role of the cerebrospinal fluid (CSF) in amyotrophic lateral sclerosis (ALS). The CSF proteome in ALS patients contains increased pro-inflammatory proteins and molecules compared to normal [1-3], possibly allowing for the spread of pathogenic proteins throughout the nervous system [4-6]. Infusion of ALS-CSF into mice over two weeks (at 0.25 ul/hr) led to TDP-43 accumulation, a decline in locomotor function, and alterations of muscle tissue [6].

We hypothesized that CSF filtration could remove pathogenic proteins and reduce motor phenotypes in ALS. Filtration was explored in the 1990s using a method called liquorpheresis to filter and reintroduce CSF into the thecal sac with a syringe pump [7,8]. Small trials of liquorpheresis showed subjective improvement in patient strength, but no difference in lung capacity or nerve conduction studies [7,8]. Studies on Guillain-Barré syndrome found that liquorpheresis restored nerve function and aided patient recovery [9,10]. Researchers speculated filtration of inflammatory proteins such as C5a, IFN-gamma, IL-2, and IL-6, but lacked studies confirming this [7,8]. Recently, another method of filtration of ALS-CSF was shown to prevent motor defects and motor neuron death when injected into mice [11].

One newer method to filter CSF is Neurapheresis™, which extracts CSF from the lumbar subarachnoid space using a dual-lumen catheter, filters pathogenic material, and reintroduces CSF into the midthoracic region [12]. Neurapheresis has a variety of processing paradigms, with 100 kilodalton (kD) filtration being studied most extensively and shown to remove proteins by size, collect waste for further study, and can tightly control filtration rate and pressures [13]. Neurapheresis was effective in depleting *Cryptococcus neoformans* from rabbits in vivo [12] and VX2 carcinoma cells in vitro [14] and has demonstrated success over conventional lumbar drains in removing blood from CSF for post-subarachnoid hemorrhage patients [15].

Neurapheresis has been demonstrated as a therapeutic option for subarachnoid hemorrhage with minimal adverse effects in patients [16], thus could be a safe and effective option for ALS patients. To test the hypothesis that filtration could alter the proteome of ALS-CSF and reduce its pathogenic capability, we filtered the CSF from patients with ALS using ex-vivo centrifugal filtration and Neurapheresis, analyzed its proteome with mass spectrometry, and injected it into mice to determine its phenotypic effect. Here we present our findings demonstrating changes in the proteome of ALS-CSF after filtration and altered motor activity in mice injected with ALS-CSF.

## Materials and methods

CSF samples

Ten ALS patient CSF samples were procured from the Department of Veteran Affairs, Biorepository Brain Bank Biobank (VABBB), Boston, Massachusetts, United States, approved by Duke University Institutional Review Board (approval number: Pro00102163), and one sample was collected from an ALS patient at Duke University Medical Center, approved by Duke University Institutional Review Board (approval number: Pro00100316), via lumbar puncture with informed consent in accordance with the Duke University Medical Center Institutional Review Board. Demographic information, including patient age and sex of the samples, are shown in Table 1. The age of onset and type of ALS (bulbar vs limb) was not available for the samples collected from the VABBB, but the patient from Duke University Medical Center had bulbar-onset ALS at the age of 42 years. None of the patients had a family history of ALS. One normal human CSF sample from a commercial biorepository was used as a control for proteomics analysis. 

**Table 1:** Characteristics of CSF samples from patients with ALS Biobank 1-10 were collected from the Department of Veteran Affairs Biorepository Brain Bank, Boston, Massachusetts Duke Patient Sample was collected from a patient at Duke University Medical Center

CSF Sample Designation	Sex	Age at Sample Collection
Biobank 1	M	62
Biobank 2	M	74
Biobank 3	M	79
Biobank 4	M	66
Biobank 5	M	69
Biobank 6	M	75
Biobank 7	M	68
Biobank 8	M	68
Biobank 9	M	87
Biobank 10	M	85
Duke Patient Sample	M	42

CSF filtration

CSF fractions from 11 samples were used in the proteomics study, of which 10 were filtered. The control was not filtered. Biorepository samples were stored at -80°C, thawed on ice, and then filtered according to the manufacturer’s instructions via 100kD ultra-centrifugation filters (Amicon®, Merck KGaA, Darmstadt, Germany). The Duke patient sample was maintained at room temperature after collection and filtered using Neurapheresis with the modification that a single 100kD tangential flow filter was used instead of the dual filter design used in clinical trials. This sample was used in the mouse movement experiments. Processed samples were stored at -80°C until analysis.

Mass spectrometry proteomics

The volume necessary to reach 50µg total protein was calculated from the bicinchoninic acid assay [17] result of the unfiltered CSF fraction from each patient, and each respective filtered sample used this volume. CSF was dried in a centrifugal concentrator and reconstituted in 60µL, 50mM NH4HCO3, 0.1% RapiGest™ SF solution (Waters Corporation, Milford, Massachusetts, United States). After denaturing proteins at 95oC for five minutes, samples were incubated for 30 minutes at 60^o^C with 6µL, 50mM dithiothreitol, then incubated in the dark for 30 minutes with 4µL 250mM iodoacetamide. Alkylation was quenched with 6µL dithiothreitol. Samples were incubated at 37^o^C for four hours with 14µL trypsin (2µg). Digestion was quenched with 10µL 5% formic acid.

Peptide digests were analyzed with an Easy-nLC™ 1200 system (Thermo Fisher Scientific Inc., Waltham, Massachusetts, United States) interfaced with an Orbitrap Exploris™ 480 Mass Spectrometer (Thermo Fisher Scientific Inc.). A quantity of 2µL was injected on an Acclaim™ PepMap™ (Thermo Fisher Scientific Inc.) 100 C18 LC trap column (0.075mm×20mm, 3µm particle) in line with an EASY-Spray™ (Thermo Fisher Scientific Inc.) analytical column (0.075mm×250mm, 2µm particle, C18) at 45^o^C. Mobile phases were water containing 2% acetonitrile, 0.1% formic acid (mobile phase A), and acetonitrile containing 20% water and 0.1% formic acid (mobile phase B(MPB)). MPB was held at 5% for two minutes, increased to 25% over 47 minutes, increased to 40% over eight minutes, increased to 95% in one minute, and held at 95% for 16 minutes. Mass spectrometer parameters were: 1.9kV positive mode spray voltage; ion transfer tube temperature, 275°C; master scan cycle time, 1.5 seconds; m/z scan range, 375 to 1,600 at 120K resolution; 300% normalized automatic gain control; 120ms maximum full scan injection time; radio frequency lens, 40%; 15K mass resolving power for data-dependent scans; 1.5m/z isolation window; 30% normalized higher-energy collisional dissociation; 100% normalized Automatic Gain Control (AGC) Target; 21ms maximum injection time; and dynamic exclusion applied for 20-second periods.

Proteome Discoverer 2.4.305 (Thermo Fisher Scientific Inc.) was used to interrogate raw data against a *Homo sapiens *protein database (Taxon 9606) obtained from UniProtKB/Swiss-Prot (42,253 sequences) and calculate protein abundances. SEQUEST-HT search node used the following parameters: tryptic cleavage at arginine and lysine, maximum of three missed cleavage sites; minimum peptide length of six amino acids; 5ppm precursor mass tolerance; 0.02Da fragment mass tolerance; maximum of three equal and four total dynamic modifications, which were oxidation of methionine and deamidation of asparagine and glutamine; dynamic protein terminus modifications of acetyl addition or methionine loss; and static carbamidomethylation of cysteine. Peptides were validated by the Percolator node with q-value set to 0.05 and strict false discovery rate set to 0.01. Protein abundances were calculated using precursor ion intensities and the summed abundances of precursors without normalization or scaling.

Mouse injections and experiments

A total of 36 mice were divided into groups of 12 for unfiltered CSF, filtered CSF, and control (saline), respectively. A quantity of 5ul CSF was injected into the Cisterna Magna (ICM) every six days for three injections per mouse. For pain management, buprenorphine was injected subcutaneously at 0.5mg/Kg before ICM [18]. Mice were anesthetized with 2% isoflurane. The head was secured in a stereotaxic frame and the skin was cleaned with ethanol. After skin incision, the muscle was retracted, and the cisterna magna exposed. Using a 5ul Hamilton syringe (Hamilton, Reno, Nevada, United States) with a 33G needle, CSF was injected in the subarachnoid space at 1ul/min. 

Mice were tested in the open field and the ability to dismount a vertical pole. Pole tests were performed on days 1, 1.25, and 10 after the final injection. The open field test was performed on days 1 and 10 post-injection. For the pole test [19], the time to dismount a vertical pole was recorded by an observer. For the open field test [20], a 50x50cm arena was placed under a recording camera and lit by a ceiling light. The mice were placed in a corner and given 20 minutes to explore. The total distance traveled over twenty minutes was recorded using the CleverSys TopScan video tracking system (CleverSys Inc., Reston, Virginia, United States).

Statistical analysis

Data were analyzed and plotted using R 4.1.1 (RStudio, Boston, Massachusetts, United States) [21] and GraphPad Prism 9.3.1 (Dotmatics, Boston, Massachusetts, United States) [22]. Data were grouped into control, filtered, and unfiltered. Within groups, raw abundance values were aggregated by protein, and summary statistics were calculated. Samples without a given protein were excluded from analysis for that protein. Key proteins identified as biomarkers in ALS were selected for further analysis [1,2,6,23-27]. Differences in abundances between groups were calculated using Welch’s t-test. For groups with fewer than four observations, the protein was excluded from analysis. Bonferroni correction for 48 comparisons was applied to set alpha at 1.04E-3.

Mean time to descend in the pole test and mean distance traveled in the open field were calculated for each group at each time point. Two-way multiple comparisons ANOVA was used to any significant differences between experimental groups set at alpha=0.05, with post-hoc analysis done with multiple comparisons testing at alpha=0.05.

## Results

Proteomics

**Figure 1:**
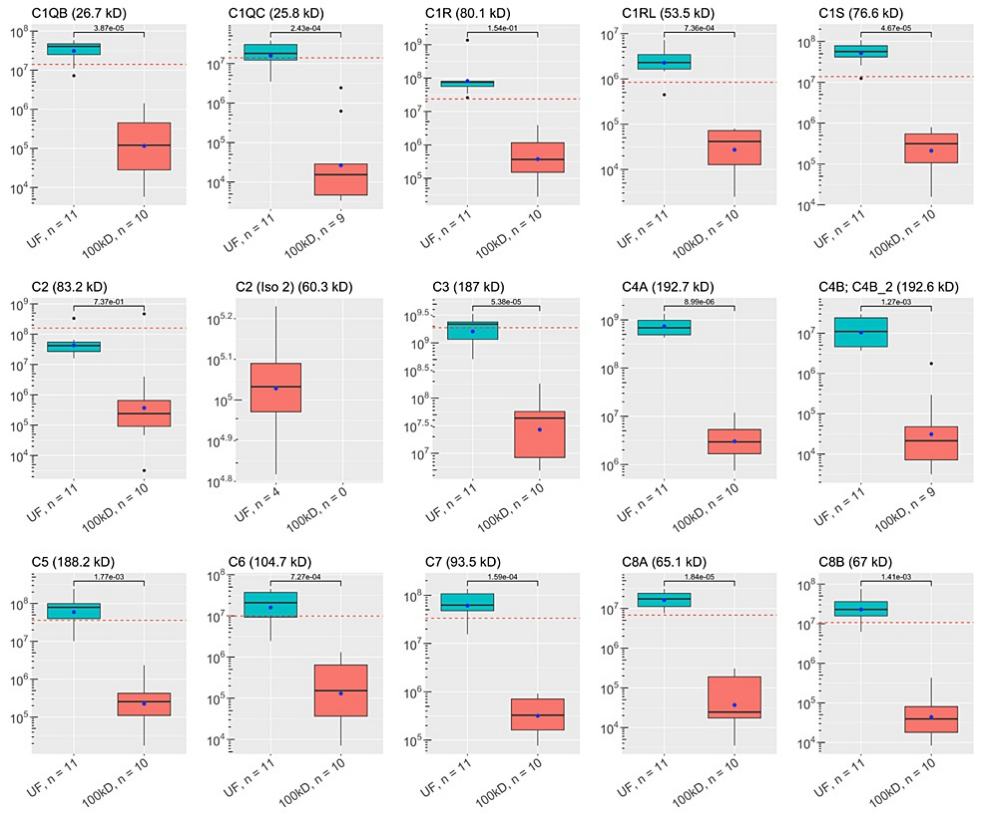
Abundances of key ALS proteins before and after CSF filtration: complement system proteins These plots visualize the abundances of key proteins in samples of each experimental group as analyzed by discovery proteomics. Each box-and-whisker barplot shows the median, Q1, Q3, standard deviation as error bars, and mean as a point. The abundance of proteins discovered in the single control sample are included as red dashed lines in relevant graphs. The molecular weight of each protein is included in the title of each plot. The y-axis shows abundance values on a log scale. The number of samples with protein identified is also included with the x-labels for each plot. P-values are shown, indicating results for Welch’s t-test comparisons between groups. UF: unfiltered ALS-CSF; 100kD: 100kD filtered ALS-CSF; ALS: amyotrophic lateral sclerosis; CSF: cerebrospinal fluid

A total of 1,648 proteins were identified in the ALS-CSF samples (shown in Appendices). Of these, 58 were identified as key proteins for further analysis. [1,2,6,23-27]. The average abundances and number of samples containing each protein in the unfiltered and filtered CSF can be found in Figure 1**.** Key proteins were grouped by functionality: there were 29 complement proteins, four serine protease inhibitors (SERPINs), three chitinases, 11 neurofilaments, tau proteins, and neuroendocrine proteins, and 11 other immune proteins.

**Figure 2:**
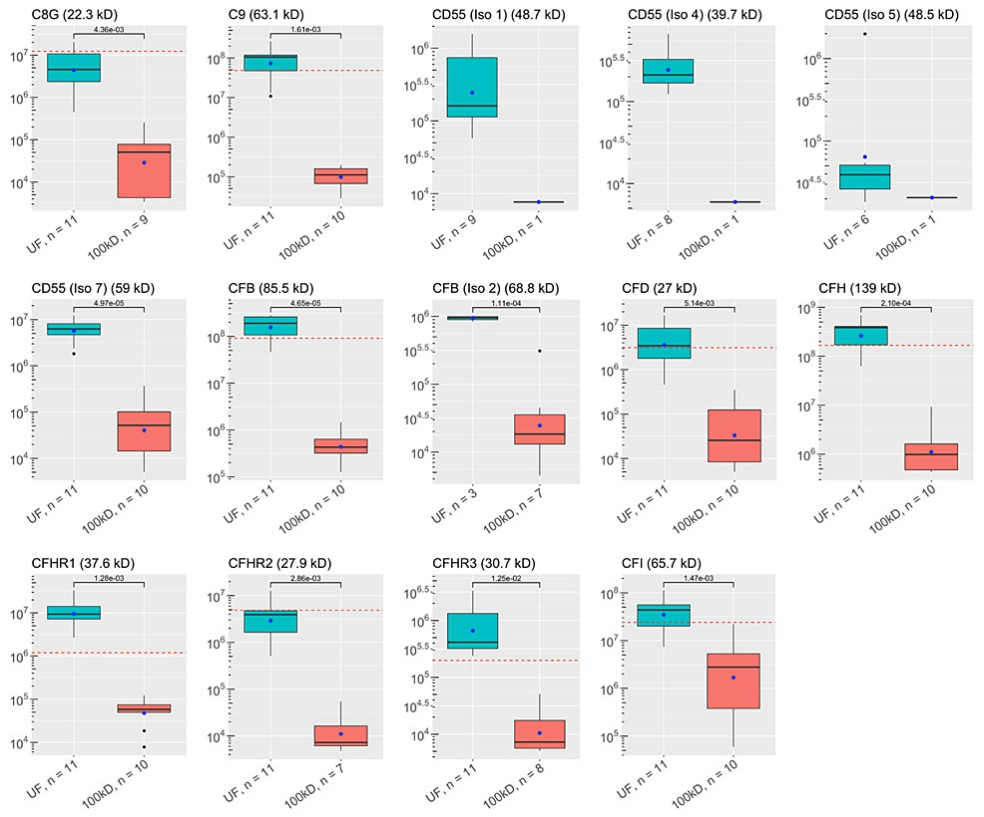
Abundances of key ALS proteins before and after CSF filtration: more complement system proteins These plots visualize the abundances of key proteins in samples of each experimental group as analyzed by discovery proteomics. Each box-and-whisker barplot shows the median, Q1, Q3, standard deviation as error bars, and mean as a point. The abundance of proteins discovered in the single control sample are included as red dashed lines in relevant graphs. The molecular weight of each protein is included in the title of each plot. The y-axis shows abundance values on a log-scale. The number of samples with protein identified is also included with the x-labels for each plot. P-values are shown, indicating results for Welch’s t-test comparisons between groups. UF: unfiltered ALS-CSF; 100kD: 100kD filtered ALS-CSF; ALS: amyotrophic lateral sclerosis; CSF: cerebrospinal fluid

**Figure 3:**
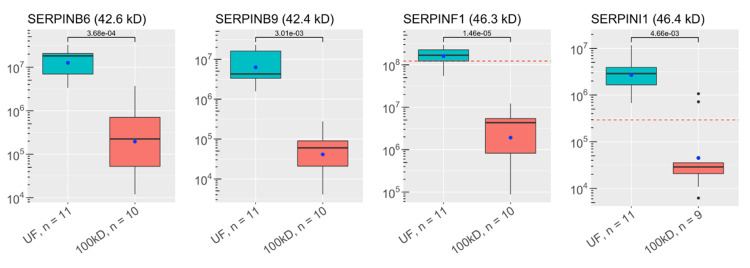
Abundances of key ALS proteins before and after CSF filtration: serine protein inhibitors (SERPINs) These plots visualize the abundances of key proteins in samples of each experimental group as analyzed by discovery proteomics. Each box-and-whisker barplot shows the median, Q1, Q3, standard deviation as error bars, and mean as a point. The abundance of proteins discovered in the single control sample are included as red dashed lines in relevant graphs. The molecular weight of each protein is included in the title of each plot. The y-axis shows abundance values on a log-scale. The number of samples with protein identified is also included with the x-labels for each plot. P-values are shown, indicating results for Welch’s t-test comparisons between groups. UF: unfiltered ALS-CSF; 100kD: 100kD filtered ALS-CSF; ALS: amyotrophic lateral sclerosis; CSF: cerebrospinal fluid

**Figure 4:**
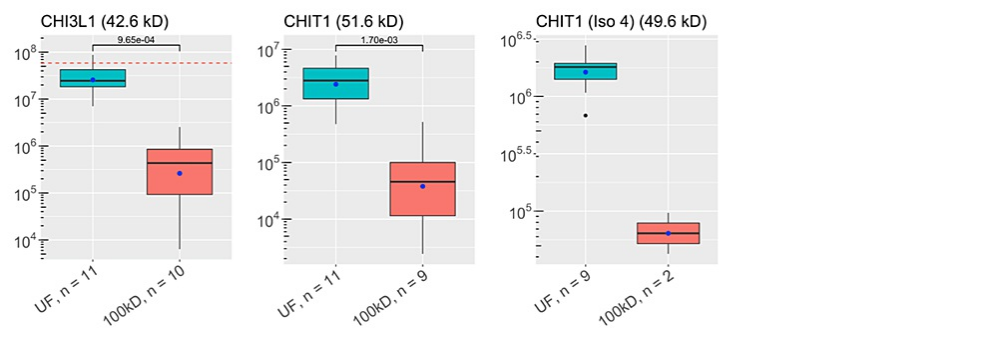
Abundances of key ALS proteins before and after CSF filtration: chitinases These plots visualize the abundances of key proteins in samples of each experimental group as analyzed by discovery proteomics. Each box-and-whisker barplot shows the median, Q1, Q3, standard deviation as error bars, and mean as a point. The abundance of proteins discovered in the single control sample are included as red dashed lines in relevant graphs. The molecular weight of each protein is included in the title of each plot. The y-axis shows abundance values on a log-scale. The number of samples with protein identified is also included with the x-labels for each plot. P-values are shown, indicating results for Welch’s t-test comparisons between groups. UF: unfiltered ALS-CSF; 100kD: 100kD filtered ALS-CSF; ALS: amyotrophic lateral sclerosis; CSF: cerebrospinal fluid

**Figure 5:**
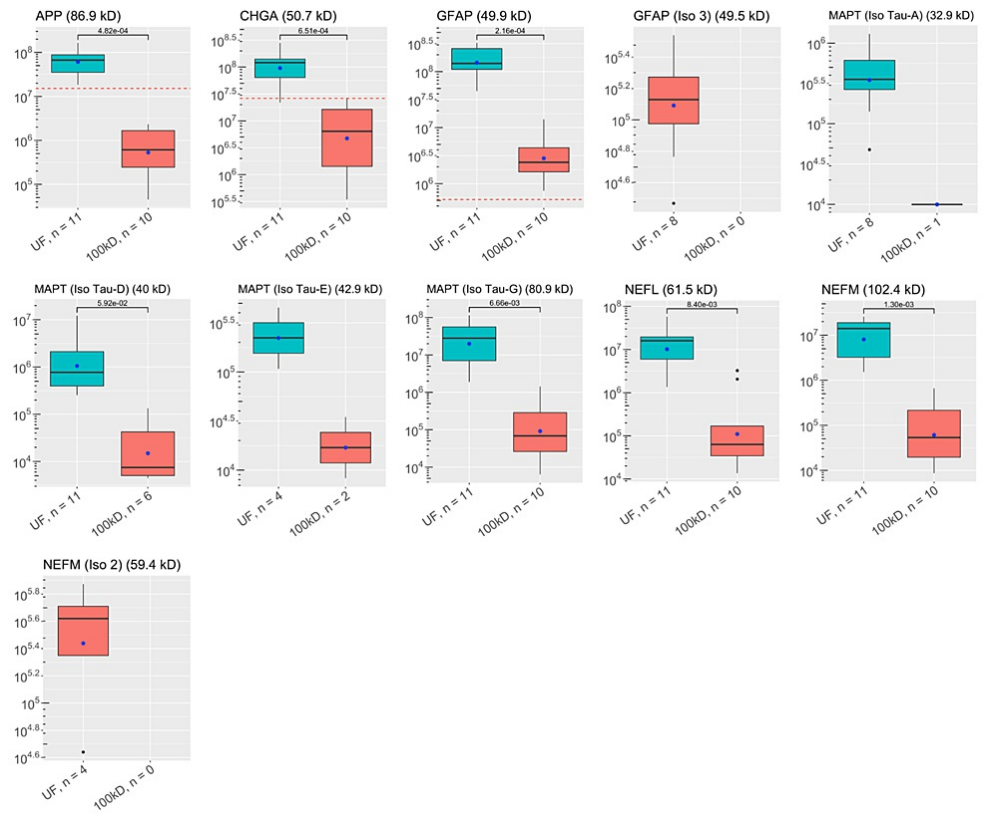
Abundances of key ALS proteins before and after CSF filtration: neurofilaments, Tau proteins, and other neurological proteins These plots visualize the abundances of key proteins in samples of each experimental group as analyzed by discovery proteomics. Each box-and-whisker barplot shows the median, Q1, Q3, standard deviation as error bars, and mean as a point. The abundance of proteins discovered in the single control sample are included as red dashed lines in relevant graphs. The molecular weight of each protein is included in the title of each plot. The y-axis shows abundance values on a log-scale. The number of samples with protein identified is also included with the x-labels for each plot. P-values are shown, indicating results for Welch’s t-test comparisons between groups. UF: unfiltered ALS-CSF; 100kD: 100kD filtered ALS-CSF; ALS: amyotrophic lateral sclerosis; CSF: cerebrospinal fluid

**Figure 6:**
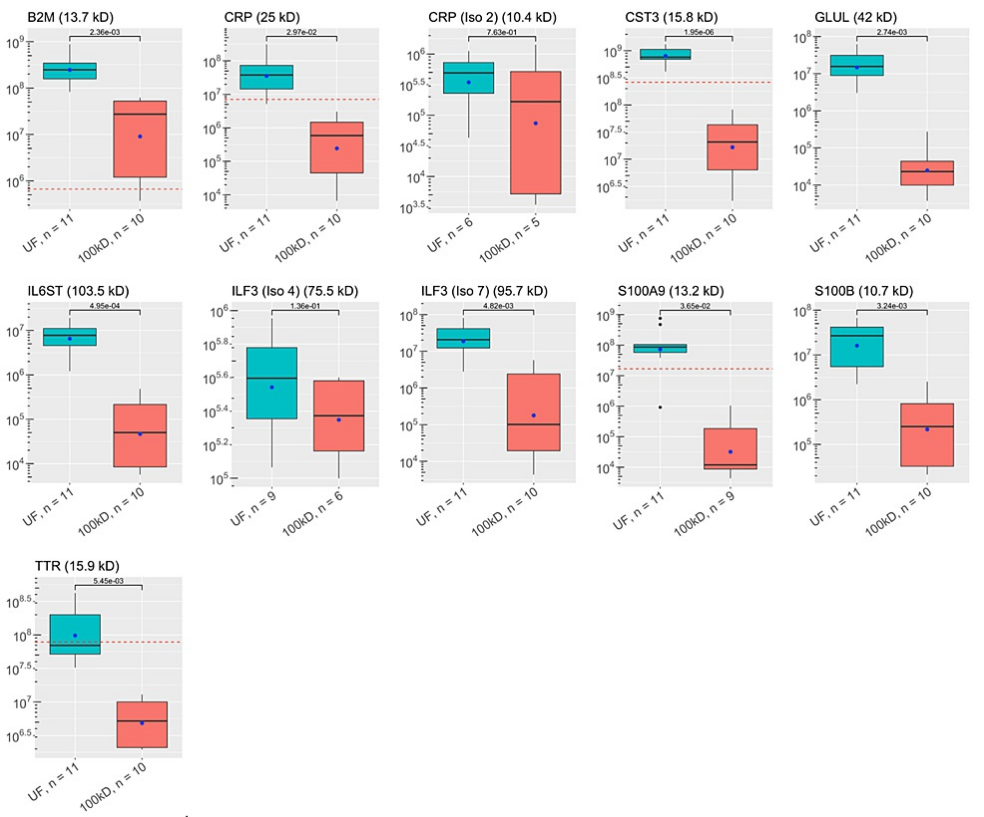
Abundances of key ALS proteins before and after CSF filtration: other inflammatory proteins These plots visualize the abundances of key proteins in samples of each experimental group as analyzed by discovery proteomics. Each box-and-whisker barplot shows the median, Q1, Q3, standard deviation as error bars, and mean as a point. The abundance of proteins discovered in the single control sample are included as red dashed lines in relevant graphs. The molecular weight of each protein is included in the title of each plot. The y-axis shows abundance values on a log-scale. The number of samples with protein identified is also included with the x-labels for each plot. P-values are shown, indicating results for Welch’s t-test comparisons between groups. UF: unfiltered ALS-CSF; 100kD: 100kD filtered ALS-CSF; ALS: amyotrophic lateral sclerosis; CSF: cerebrospinal fluid

All proteins had lower average abundances when filtered, but 13 complement proteins, two SERPINs, one chitinase, three “neuro-proteins”, and four other immunological proteins were statistically significantly less abundant when filtered. Comparisons between the groups are shown in Figure 1. Four complement proteins, one chitinase, and three “neuro-proteins” were not found in enough filtered samples to qualify for statistical testing. Thirty-two of the key proteins were identified in the control sample. Statistical comparisons between the control sample and the experimental samples were not done since there was a single control. The abundances of the control sample are shown in Figures 1-6.

Mouse motor tests

The pole descent test assessed maneuverability, coordination, and fine motor control [19]. Mice were expected to descend quicker with repeated trials. Only 11 of the 12 control mice performed the pole test. On day 1, the control mice descended at an average of 14.86 seconds, filtered mice at 16.43 seconds, and unfiltered mice at 16.83 seconds; on day 1.25, at an average of 10.61 seconds, 11.64 seconds, and 12.75 seconds, respectively; on day 10, at 10.06 seconds, 11.14 seconds, and 14.25 seconds, respectively. Two-way ANOVA between groups was significant at p=0.04. Post-hoc analysis showed that at day 10, unfiltered mice took significantly longer to descend the pole (p=0.02).

**Figure 7:**
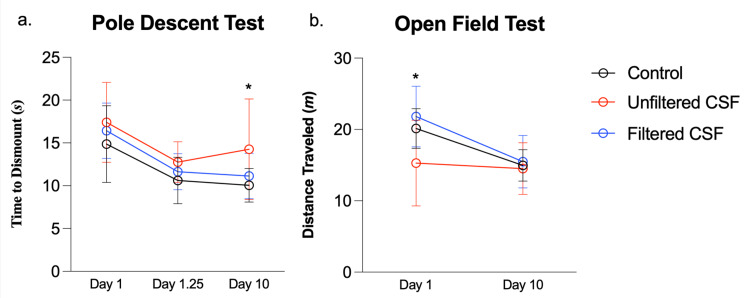
Mouse behavioral studies These plots visual the results from (a) pole descent tests and (b) open field tests that were conducted with mice after injection with either saline (control), 100kD filtered ALS-CSF, or unfiltered CSF. a) The results of the pole descent test over time are shown as the mean time to dismount the pole, in seconds, for each experimental group over successive trials. The error bars represent standard deviation. b) The results of the open field test over time are shown as the mean distance traveled in 20 minutes, in meters, for each experimental group over successive trials. The error bars represent standard deviation. ALS: amyotrophic lateral sclerosis; CSF: cerebrospinal fluid

The open field test assessed gross motor activity [20], and it is said that mice typically explore the environment when placed in the open field [28]. The control mice, filtered mice, and unfiltered mice traveled an average of 19.95m, 21.81m, and 16.83m, respectively, on day 1, and 15.15m, 15.149m, and 14.52m, respectively, on day 10. Two-factor repeated measures ANOVA was significant at p=0.02, thus post-hoc analysis was conducted. On day 1, the filtered group traveled significantly further than the unfiltered group (p=0.0004). The control group also traveled further than the unfiltered group (p=0.01). None of the groups were significantly different at 10 days. Results are shown in Table 2 and Figure 7.

**Table 2:** Results of mouse behavioral studies This table outlines the results from pole descent tests and open field tests that were conducted with mice after injection with either saline (control), 100kD filtered ALS-CSF, or unfiltered CSF. Repeated measures two-factor ANOVA with alpha set at 0.05 was conducted to determine if any differences were detected between groups or over time. Post-hoc analysis was done via Welch’s t-test to determine differences between experimental groups at each time point, with alpha set at 0.05. ALS: amyotrophic lateral sclerosis; CSF: cerebrospinal fluid

	Control (n=11)	Filtered (n=12)	Unfiltered (n=12)	Control vs Filtered	Control vs Unfiltered	Filtered vs Unfiltered
Pole Test (mean time to dismount +/- SD, s) Two-way ANOVA between groups: p = 0.04						
Day 1	14.86 +/- 4.47	16.43 +/-​​​​​​​ 3.23	16.83 +/-​​​​​​​ 4.67	p=0.55	p=0.21	p=0.79
Day 1.25	10.61 +/-​​​​​​​ 2.71	11.64 +/-​​​​​​​ 2.11	12.75 +/-​​​​​​​ 2.38	p=0.77	p=0.33	p=0.73
Day 10	10.06 +/-​​​​​​​ 1.96	11.14 +/-​​​​​​​ 2.65	14.25 +/- 5.89	p=0.75	p=0.02*	p=0.09
Open Field Test (mean distance traveled SD, m) Two-way ANOVA between groups: p = 0.02						
Day 1	19.95 +/-​​​​​​​ 2.78 (n=12)	21.81 +/-​​​​​​​ 4.26	15.29 +/-​​​​​​​ 6.01	p=0.66	p=0.01*	p=0.0004*
Day 10	15.15 +/-​​​​​​​ 2.21 (n=12)	15.49 +/-​​​​​​​ 3.68	14.52 +/-​​​​​​​ 3.62	p=0.98	p=0.99	p=0.98

## Discussion

We analyzed the proteome of the CSF from patients with ALS, characterized the changes in composition after filtration, and tested the effect of ALS-CSF on mouse motor activity. Proteomics identified over 1,600 proteins in the CSF, of which 58 were selected as key proteins implicated in the disease [1,2,6,23-27]. This even results in the filtration of other proteins (mentioned in Table in appendices). However, as previously discussed, Neurapheresis is a safe treatment for cryptococcal meningitis and subarachnoid hemorrhage, so this does not appear to be a detriment. Interestingly, even though we used 100kD filters, many proteins under 100kD in size were significantly reduced in abundance, likely due to clustering and aggregation of proteins that occur in body fluids. Here, we discuss the implications of the identified proteins in the disease process of ALS.

Key proteins

The complement proteins mark targets for phagocytosis and elimination [29]. Excessive complement in neurons of ALS patients is associated with neuronal and glial degeneration [30]. C5aR1 inhibitors reduce motor deficits and increase survival time in rodent ALS models [31,32]. If complement inhibition improves ALS phenotypes, filtration of these out of the CSF could be beneficial to patients. The accumulation of misfolded SERPINs has been found in the tissue of brains affected by ALS [33] and in the CSF mouse models [34], but it is unclear if misfolding drives ALS or is a result of ALS. SERPINF1 has been shown to have a protective effect on motor neurons in vitro, leading to speculation that its increase in the CSF in ALS is a protective mechanism [35]. Given the lack of in-vivo studies, we cannot speculate if filtering out SERPINF1 has a negative effect on ALS progression. SERPINI1 is involved in neurogenesis and dendritic maturation, but increases have been associated with neurodegeneration in Alzheimer’s [36]. Although no explicit link has been made with ALS, perhaps a similar neurodegenerative process occurs in ALS. Chitinases are produced by astrocytes and microglia and play a role in the nervous system’s immune response [37]. CHIT1, CHI3L1, and CHI3L2 have been found to be elevated in the CSF of patients with ALS, with levels correlating with disease progression [37-42].

In-vivo studies have directly linked chromogranin A (CHGA) to ALS, demonstrating it promotes secretion of SOD1, leading to neuron degeneration [43,44]. Glial fibrillary acidic protein (GFAP) is known to increase during the process of gliosis and astrocyte-driven inflammation in the brain [45]. Amyloid precursor protein (APP) has been implicated in Alzheimer’s, but its role in ALS is less clear. TDP-43, a key protein implicated in ALS, is involved in the splicing of APP [46]. Perhaps in ALS, APP accumulation is a result of neuronal damage rather than a causative agent. The MAPT, or Tau proteins, which have been associated with the formation of neurofibrillary tangles [47], and neurofilament chains (NEFL, NEFM, and NFH) [48] are increased in the CSF of ALS patients and correlate with disease severity, thus could be used as biomarkers to track disease [47-53]. Reducing levels of pro-inflammatory proteins such as CHGA and GFAP could be therapeutic for ALS and biomarkers such as APP, MAPT, and neurofilaments could confirm if filtration is modifying ALS progression meaningfully.

C-reactive protein (CRP) may contribute to damage in Alzheimer’s [54], and a strong negative correlation between CRP level in ALS-CSF and clinical outcomes has been demonstrated [55]. In an ALS mouse model, there was a significant increase in GS+ cells (Glutamate synthase, encoded by GLUL), correlated with microglial activation, indicating possible mechanisms of inflammation [56-58]. ILF3 antisense RNA 1 (ILF3-AS1), while not studied in ALS, leads to increased IL-6 and TNF-a in astrocytes, indicating a potential role in inflammation [59]. Transthyretin (TTR) was shown to be dysregulated in ALS compared to control spinal cord samples [60] and Beta 2-microglobulin (B2M) has shown differential RNA expression in the peripheral white blood cells of ALS patients compared to control [61], making them potential biomarkers.

S100-A9 mRNA levels were increased at end-stage ALS but deleting it in a mouse model had no impact on motor neuron survival and slightly accelerated symptoms [62]. In one study, S100B was found to be upregulated in ALS spinal cord samples [63], but in another, levels decreased with disease progression [64]. Genotypic frequencies of CST3 were not statistically significant in ALS patients compared to controls [65], suggesting no expressional difference in cystatin C. The roles of S100-A9, S100B, and CST3 are still unclear in ALS, thus they should be studied further.

Mouse motor tests

The open field measures gross locomotor activity and has been previously studied in the context of ALS [6,66]. On day 1, the unfiltered group traveled significantly less than the filtered control groups, suggesting a motor deficit could have resulted from the ALS-CSF to prevent exploration, but all groups traveled similar distances on day 10. In the open field, mice naturally explore a novel environment [67]; thus, we can hypothesize that unfiltered CSF hampered the tendency of mice to move around the open field on day 1. By day 10, the other two groups naturally reduced locomotion due to the lack of novelty, but the unfiltered group was still unable to explore.

The pole descent test has been tested as a measure of Parkinson’s disease [19,68], but not ALS. All three mouse groups decreased time to descend between the first and second trials, the control and filtered CSF groups decreased their descent time a little more by day 10. The unfiltered group did show an increase in average descent time between day 1.25 and day 10 and had a significantly longer descent time than the control group at this timepoint. This suggests some change in the fine motor coordination due to ALS-CSF, resulting in decreased ability to quickly maneuver. The differences in fine motor control are only apparent on day 10, while differences in the open field are apparent on day 1, suggesting differential effects on these two aspects of behavior.

Limitations

One key limitation of this study is the underlying assumption that the alteration in mouse behavior might be due to key proteins that we identified here; however, other filtered cytokines and molecules could also play a role that we have not identified in this study. Additionally, by using mass spectrometry, we are restricted to analyzing relative changes in the identified proteins. We would have had to utilize methods such as enzyme-linked immunosorbent assay (ELISA) to identify specific quantities and concentrations of proteins, but we believe that our methodology allows for adequate analysis of changes in the protein makeup of the CSF samples. Our samples are also inherently biased as we were only able to procure samples from male patients; thus, we should expand our future studies to include female patient samples. A limitation of our mouse studies is that we did not conduct studies that would allow us to monitor the recovery of the mice injected with ALS-CSF. CSF is recycled multiple times daily [69], thus these mice might have recovered function as the ALS-CSF was cycled out of their systems.

## Conclusions

We analyzed the changes in the proteome of ALS-CSF before and after filtration by molecular weight and demonstrated reductions in numerous proteins implicated in ALS pathophysiology and various biomarkers. Additionally, we demonstrated the ability of the unfiltered ALS-CSF to induce gross motor deficits in mice using pole descent and open field tests and showed that filtration could prevent this deficit. Clinical trials will be needed to test the potential of Neurapheresis as a treatment for ALS. It has been shown to be a safe and effective treatment for other neurological conditions, and with the lack of effective and affordable treatments for ALS at the present time, this could be a novel solution for patients suffering from this deadly and irreversible condition. Future studies include the implementation of repetitive filtration in mice with ALS to determine the volume and frequency of CSF filtration that could serve a therapeutic purpose.

## Appendices

**Table 3:** Supplemental data table: complete list of identified proteins and grouped abundances (means) This table shows the abundances of every protein found in CSF samples of each experimental group as analyzed by discovery proteomics. Empty cells indicate that abundances could not be calculated due to the protein not being found in any samples in that experimental group. UF: unfiltered ALS-CSF; 100kD: 100kD filtered ALS-CSF; ALS: amyotrophic lateral sclerosis; CSF: cerebrospinal fluid

Gene Symbol	UF Mean	UF SD	n	100kD Mean	100kD SD	n
C3	1.85E+09	8.94E+08	11	4.74E+07	5.29E+07	10
C4A	7.92E+08	3.17E+08	11	4.33E+06	3.78E+06	10
CST3	8.43E+08	2.80E+08	11	3.05E+07	3.05E+07	10
CFH	3.28E+08	1.91E+08	11	1.93E+06	2.72E+06	10
B2M	3.06E+08	2.28E+08	11	2.80E+07	2.60E+07	10
CFB	1.83E+08	8.89E+07	11	5.71E+05	4.40E+05	10
SERPINF1	1.77E+08	7.36E+07	11	4.19E+06	3.90E+06	10
GFAP	1.72E+08	9.85E+07	11	4.42E+06	4.75E+06	10
CHGA	1.18E+08	7.35E+07	11	1.03E+07	1.04E+07	10
C9	1.08E+08	8.35E+07	11	1.15E+05	6.06E+04	10
TTR	1.37E+08	1.22E+08	11	6.30E+06	4.36E+06	10
APP	7.41E+07	4.78E+07	11	9.84E+05	8.78E+05	10
C5	8.22E+07	6.42E+07	11	5.36E+05	7.43E+05	10
C1R	1.83E+08	3.91E+08	11	8.67E+05	1.18E+06	10
C7	7.39E+07	4.16E+07	11	4.30E+05	3.19E+05	10
S100A9	1.68E+08	2.31E+08	11	1.71E+05	3.41E+05	9
C1S	5.90E+07	2.86E+07	11	3.40E+05	2.62E+05	10
CFI	4.48E+07	3.06E+07	11	5.07E+06	6.83E+06	10
C2	6.60E+07	9.06E+07	11	4.75E+07	1.48E+08	10
C1QB	3.67E+07	1.73E+07	11	3.41E+05	4.62E+05	10
CRP	7.45E+07	9.64E+07	11	8.91E+05	1.01E+06	10
CHI3L1	3.22E+07	2.26E+07	11	7.36E+05	8.65E+05	10
MAPT (Iso Tau-G)	3.64E+07	3.51E+07	11	3.00E+05	4.59E+05	10
C8B	2.96E+07	2.25E+07	11	9.73E+04	1.38E+05	10
S100B	2.68E+07	2.26E+07	11	7.05E+05	9.25E+05	10
C6	2.24E+07	1.52E+07	11	3.83E+05	4.85E+05	10
ILF3 (Iso 7)	2.88E+07	2.52E+07	11	1.51E+06	2.18E+06	10
GLUL	2.12E+07	1.78E+07	11	5.51E+04	8.39E+04	10
C1QC	1.99E+07	1.17E+07	11	3.48E+05	8.06E+05	9
C8A	1.79E+07	7.76E+06	11	1.01E+05	1.22E+05	10
SERPINB6	1.56E+07	9.45E+06	11	6.81E+05	1.14E+06	10
NEFL	1.66E+07	1.63E+07	11	5.88E+05	1.13E+06	10
C4B; C4B_2	1.40E+07	1.03E+07	11	2.43E+05	5.78E+05	9
NEFM	1.21E+07	8.94E+06	11	1.52E+05	2.11E+05	10
IL6ST	8.59E+06	5.54E+06	11	1.37E+05	1.59E+05	10
CFHR1	1.22E+07	9.13E+06	11	5.87E+04	3.22E+04	10
SERPINB9	9.38E+06	7.93E+06	11	7.83E+04	8.40E+04	10
CD55 (Iso 7)	6.53E+06	3.16E+06	11	8.82E+04	1.13E+05	10
CFD	5.91E+06	5.42E+06	11	8.87E+04	1.18E+05	10
C8G	6.93E+06	6.21E+06	11	6.80E+04	8.00E+04	9
CFHR2	4.05E+06	3.41E+06	11	1.62E+04	1.81E+04	7
SERPINI1	3.53E+06	3.04E+06	11	2.16E+05	3.94E+05	9
C1RL	2.84E+06	1.94E+06	11	4.29E+04	3.09E+04	10
CHIT1	3.29E+06	2.49E+06	11	1.02E+05	1.62E+05	9
CHIT1 (Iso 4)	1.76E+06	6.93E+05	9	6.96E+04	3.85E+04	2
CFB (Iso 2)	9.42E+05	9.53E+04	3	6.18E+04	1.10E+05	7
MAPT (Iso Tau-D)	2.37E+06	3.63E+06	11	3.83E+04	5.38E+04	6
ILF3 (Iso 4)	4.35E+05	2.87E+05	9	2.58E+05	1.39E+05	6
CFHR3	1.06E+06	1.14E+06	11	1.49E+04	1.56E+04	8
MAPT (Iso Tau-A)	5.26E+05	4.67E+05	8	9.93E+03		1
CRP (Iso 2)	5.21E+05	3.96E+05	6	4.23E+05	5.97E+05	5
NEFM (Iso 2)	4.07E+05	2.89E+05	4			0
CD55 (Iso 4)	2.80E+05	1.78E+05	8	5.95E+03		1
MAPT (Iso Tau-E)	2.54E+05	1.50E+05	4	2.15E+04	1.87E+04	2
CD55 (Iso 1)	4.41E+05	5.11E+05	9	7.68E+03		1
GFAP (Iso 3)	1.57E+05	1.07E+05	8			0
C2 (Iso 2)	1.13E+05	4.31E+04	4			0
CD55 (Iso 5)	3.61E+05	7.99E+05	6	2.06E+04		1
SCG5	1.77E+07	8.96E+06	11	1.82E+06	1.90E+06	10
SCG5; SGNE1	5.32E+05	4.07E+05	11	9.77E+03	7.59E+03	5
NTRK2	7.72E+06	5.66E+06	11	4.42E+04	6.40E+04	10
ALDH1L1			0	2.48E+05	1.53E+05	3
FN1			0	6.96E+04	9.02E+04	7
ANK2			0	3.47E+04	3.29E+04	6
PEA15			0	7.98E+04	8.16E+04	5
ADK			0	6.39E+04	1.01E+05	4
FGFR1			0	1.15E+04	5.16E+03	3
ALB	5.55E+10	1.63E+10	11	1.60E+09	6.96E+08	10
TF	4.46E+09	1.16E+09	11	1.44E+08	1.16E+08	10
SERPINA1	4.39E+09	2.96E+09	11	6.72E+07	3.50E+07	10
A2M	3.91E+09	3.16E+09	11	5.54E+07	4.69E+07	10
HP	3.42E+09	2.47E+09	11	3.67E+07	5.55E+07	10
HBA1; HBA2	4.04E+09	3.54E+09	11	6.49E+07	4.89E+07	10
HBB	3.50E+09	3.02E+09	11	5.75E+07	4.57E+07	10
APOD	1.50E+09	5.15E+08	11	1.49E+07	1.85E+07	10
PTGDS	1.70E+09	5.66E+08	11	2.35E+08	1.93E+08	10
GOT1	1.18E+09	9.45E+08	11	2.14E+07	3.17E+07	10
HPX	1.23E+09	5.81E+08	11	2.34E+07	1.41E+07	10
APOE	9.76E+08	3.05E+08	11	1.48E+06	7.49E+05	10
APOA1	9.12E+08	5.38E+08	11	6.55E+06	5.03E+06	10
SERPINA3	1.02E+09	6.68E+08	11	5.51E+06	2.61E+06	10
IGHA1	2.20E+09	2.72E+09	11	1.25E+07	1.36E+07	10
TTN	1.12E+09	7.28E+08	11	8.28E+06	9.04E+06	10
GSN	7.83E+08	3.71E+08	11	3.12E+06	4.27E+06	10
FGB	8.88E+08	6.31E+08	11	2.10E+06	1.05E+06	10
FGG	9.02E+08	6.02E+08	11	8.11E+06	1.69E+07	10
FGA	8.40E+08	5.26E+08	11	2.46E+07	2.82E+07	10
ORM1	1.01E+09	8.07E+08	11	2.11E+07	1.41E+07	10
GAPDH; HEL-S-162eP; LOC117800120	8.01E+08	5.22E+08	11	4.13E+06	1.82E+06	10
CLU	6.61E+08	2.89E+08	11	2.17E+06	1.90E+06	10
IGLL5	1.13E+09	1.12E+09	11	2.00E+07	2.07E+07	10
APOB	5.16E+08	4.13E+08	11	2.63E+06	9.74E+05	10
IGHG2	7.77E+08	7.64E+08	11	2.71E+07	2.25E+07	10
PEBP1	4.02E+08	2.50E+08	11	6.81E+07	6.75E+07	10
CP	3.63E+08	2.67E+08	11	3.86E+06	2.42E+06	10
ENO1; HEL-S-17; LOC117800423	4.32E+08	2.87E+08	11	7.49E+06	7.55E+06	10
PPIA	4.63E+08	3.08E+08	11	3.14E+07	3.12E+07	10
ACTB	4.80E+08	3.19E+08	11	2.46E+06	1.71E+06	10
MYH9	3.84E+08	3.21E+08	11	3.87E+06	3.52E+06	10
PSMC2	2.79E+08	2.99E+08	11	7.95E+05	2.03E+06	10
NEB	2.59E+08	1.20E+08	11	4.97E+06	3.82E+06	10
KRT6A	3.38E+08	2.53E+08	11	6.64E+06	7.21E+06	10
IMPA1	1.88E+08	9.98E+07	11	6.83E+05	1.28E+06	10
COTL1	3.89E+08	4.29E+08	11	3.30E+07	2.87E+07	10
AHNAK	3.51E+08	2.95E+08	11	3.84E+06	2.08E+06	10
PKM	4.28E+08	3.76E+08	11	2.23E+06	9.91E+05	10
SERPING1	2.76E+08	1.60E+08	11	1.45E+06	2.79E+06	10
SYNE2	3.56E+08	2.80E+08	11	2.25E+06	2.75E+06	10
LCN2	2.45E+08	1.65E+08	11	1.66E+05	3.22E+05	10
CKB	3.60E+08	2.29E+08	11	3.02E+06	3.02E+06	10
IGHG3	3.29E+08	3.22E+08	11	4.75E+06	3.09E+06	10
CRTAC1	2.95E+08	2.24E+08	11	2.64E+05	2.63E+05	10
SYNE1	2.64E+08	1.87E+08	11	2.72E+06	1.97E+06	10
IGHM	5.67E+08	7.85E+08	11	1.37E+06	1.08E+06	10
DST	2.24E+08	1.41E+08	11	2.13E+06	1.25E+06	10
CA1	1.89E+08	1.02E+08	11	7.97E+06	8.28E+06	10
PGK1	2.51E+08	1.93E+08	11	1.07E+07	1.26E+07	10
VIM	3.04E+08	3.02E+08	11	3.37E+06	3.81E+06	10
LDHB	2.78E+08	1.95E+08	11	2.60E+06	1.37E+06	10
AGT	1.89E+08	9.33E+07	11	3.85E+06	3.23E+06	10
IGHG4	5.22E+08	6.82E+08	11	5.04E+06	3.32E+06	10
PARK7	2.17E+08	1.14E+08	11	4.53E+06	3.37E+06	10
HBD	2.78E+08	1.91E+08	11	1.06E+06	9.77E+05	10
ITIH4	2.67E+08	2.23E+08	11	6.06E+05	4.27E+05	10
A1BG	1.89E+08	1.12E+08	11	7.37E+05	5.95E+05	10
CAP1	1.60E+08	6.29E+07	11	2.54E+05	3.79E+05	10
PLEC	1.85E+08	1.19E+08	11	2.19E+06	2.37E+06	10
SPTAN1	1.41E+08	6.76E+07	11	1.25E+06	1.04E+06	10
PRDX1	2.18E+08	1.53E+08	11	1.81E+06	2.15E+06	10
CHGB	1.63E+08	8.53E+07	11	1.62E+07	1.36E+07	10
FABP1	2.89E+08	2.16E+08	11	6.51E+05	9.36E+05	10
CRABP1	1.65E+08	1.04E+08	11	3.54E+06	3.94E+06	10
IGKC	3.00E+08	3.08E+08	11	5.96E+06	3.76E+06	10
UBA52	1.89E+08	8.93E+07	11	3.39E+07	3.38E+07	10
HEL-S-49; LOC117800142; TPI1	2.00E+08	1.41E+08	11	1.85E+07	1.54E+07	10
AP2A1	1.57E+08	6.53E+07	11	9.35E+04	1.51E+05	9
SERPINC1	1.26E+08	8.11E+07	11	2.80E+06	3.27E+06	10
POTEF	1.84E+08	1.37E+08	11	7.96E+05	7.13E+05	10
DKK3	1.43E+08	6.01E+07	11	4.40E+05	6.84E+05	10
SPP1	1.61E+08	9.08E+07	11	8.94E+06	8.40E+06	10
PAM	3.18E+08	2.50E+08	11	7.04E+05	8.58E+05	10
GSTP1	2.47E+08	2.27E+08	11	7.19E+06	9.09E+06	10
KARS1	1.35E+08	7.99E+07	11	2.46E+05	2.61E+05	10
AZGP1	1.67E+08	1.41E+08	11	2.51E+06	1.90E+06	10
MACF1	1.56E+08	1.27E+08	11	4.65E+06	4.23E+06	10
PRDX6	1.71E+08	1.11E+08	11	6.28E+05	7.53E+05	10
CA2	2.61E+08	2.29E+08	11	2.87E+07	4.23E+07	10
MDH1	1.46E+08	7.42E+07	11	2.09E+06	2.32E+06	10
ORM2	1.36E+08	9.05E+07	11	2.26E+06	1.87E+06	10
PLG	1.25E+08	6.71E+07	11	2.04E+06	3.30E+06	10
ALDOA	2.05E+08	1.51E+08	11	1.30E+06	1.01E+06	10
NSF	2.00E+08	1.65E+08	11	6.23E+06	6.23E+06	10
VGF	1.43E+08	7.86E+07	11	2.17E+06	2.00E+06	10
OLFML3	1.82E+08	1.56E+08	11	2.39E+06	2.27E+06	10
ASL	1.30E+08	8.86E+07	11	5.82E+04	5.02E+04	10
SELENBP1	2.15E+08	1.66E+08	11	5.98E+06	5.82E+06	10
KRT6C	1.77E+08	1.56E+08	11	5.13E+05	9.15E+05	8
NCAM1	1.77E+08	1.50E+08	11	2.86E+06	2.30E+06	10
SPTBN1	1.63E+08	1.14E+08	11	4.88E+06	3.40E+06	10
ALDOC	1.76E+08	1.86E+08	11	6.00E+05	3.25E+05	10
Cltc; CLTC	1.25E+08	4.22E+07	11	1.34E+06	9.59E+05	10
ADH1B	1.65E+08	1.28E+08	11	4.82E+05	8.36E+05	10
APOH	1.18E+08	6.23E+07	11	2.50E+05	1.92E+05	10
SPARCL1	1.07E+08	6.58E+07	11	5.02E+06	2.20E+06	10
SCG2	1.22E+08	7.67E+07	11	3.33E+06	2.75E+06	10
CRK; HEL2; LOC101343175; LOC111151387; Ywhae; YWHAE	1.51E+08	1.13E+08	11	9.40E+05	7.59E+05	10
LDHA	1.14E+08	5.97E+07	11	5.04E+05	4.43E+05	10
CBR1	1.47E+08	1.35E+08	11	4.42E+06	4.83E+06	10
KRT1	1.12E+08	3.85E+07	11	3.48E+07	3.83E+07	10
CNDP1	1.03E+08	2.84E+07	11	2.86E+06	3.32E+06	10
COL6A3	9.70E+07	4.46E+07	11	8.53E+05	1.09E+06	10
TKT	1.13E+08	6.75E+07	11	3.38E+05	3.17E+05	10
COL1A1	3.31E+08	6.56E+08	11	6.56E+06	6.39E+06	10
RAD23B	1.40E+08	9.85E+07	11	9.61E+05	1.06E+06	9
SPTBN2	9.74E+07	7.78E+07	11	1.74E+06	1.36E+06	10
SOD1	1.14E+08	6.73E+07	11	1.23E+07	1.26E+07	10
ALDH1L1	2.04E+08	2.07E+08	11	6.07E+06	6.86E+06	10
DYNC1H1	1.02E+08	4.39E+07	11	7.01E+06	1.21E+07	10
PSAT1	4.82E+08	5.26E+08	11	2.03E+05	2.72E+05	10
KNG1	9.71E+07	6.91E+07	11	3.49E+05	2.62E+05	10
NRCAM	9.97E+07	4.96E+07	11	2.47E+05	1.66E+05	10
MYH10	1.59E+08	1.20E+08	11	6.24E+05	7.02E+05	10
CPN1	1.11E+08	1.00E+08	11	1.85E+04	1.00E+04	10
ANXA2	8.12E+07	4.65E+07	11	1.65E+05	1.59E+05	10
CHL1	8.51E+07	4.55E+07	11	5.08E+05	6.40E+05	10
FCGBP	1.11E+08	9.37E+07	11	4.01E+06	5.48E+06	10
SERPIND1	1.00E+08	6.34E+07	11	1.05E+05	9.01E+04	10
PLTP	9.10E+07	5.94E+07	11	1.27E+05	1.48E+05	10
CA3	7.88E+07	6.59E+07	11	1.19E+06	1.77E+06	10
B4GAT1	8.04E+07	2.47E+07	11	2.84E+05	2.01E+05	10
ATP1A2	8.52E+07	3.19E+07	11	9.98E+05	1.05E+06	10
AKAP9	8.39E+07	3.38E+07	11	1.12E+06	1.02E+06	10
KTN1	1.08E+08	1.05E+08	11	8.14E+05	8.07E+05	10
LRG1	1.58E+08	1.36E+08	11	1.16E+06	1.31E+06	10
HNRNPU	1.11E+08	7.58E+07	11	6.54E+05	9.08E+05	10
ARF3	9.74E+07	6.38E+07	11	2.12E+04	1.99E+04	7
CAPS	1.16E+08	1.30E+08	11	3.78E+06	4.39E+06	10
PFKP	1.57E+08	1.87E+08	11	4.65E+05	8.65E+05	10
ENO2	7.25E+07	4.80E+07	11	1.24E+06	2.60E+06	10
APLP1	6.62E+07	2.96E+07	11	1.25E+06	1.27E+06	10
CLIC6	8.41E+07	6.87E+07	11	3.93E+05	4.18E+05	10
DSP	7.74E+07	2.97E+07	11	2.78E+06	4.32E+06	10
IQGAP1	6.64E+07	3.29E+07	11	4.70E+05	3.32E+05	10
CNTN1	6.66E+07	3.40E+07	11	1.08E+06	8.45E+05	10
DPYSL2	1.14E+08	8.31E+07	11	5.93E+05	4.27E+05	10
MSN	1.06E+08	6.01E+07	11	1.12E+06	8.24E+05	10
F2	7.27E+07	5.09E+07	11	1.47E+06	1.81E+06	10
UCHL1	1.08E+08	8.94E+07	11	1.03E+07	1.25E+07	10
CALB1	1.03E+08	8.31E+07	11	3.90E+06	4.40E+06	10
PGAM1	1.03E+08	7.16E+07	11	1.03E+06	9.91E+05	10
PRDX2	9.30E+07	5.45E+07	11	3.83E+05	4.45E+05	10
KRT14	7.27E+07	2.88E+07	11	8.76E+06	7.27E+06	10
COL12A1	6.08E+07	4.45E+07	11	1.93E+06	2.03E+06	10
PSMD11	8.03E+07	3.70E+07	11	5.02E+04	4.90E+04	10
ITIH2	8.85E+07	5.03E+07	11	1.98E+06	4.65E+06	10
NPEPPS	7.34E+07	4.75E+07	11	6.54E+05	1.10E+06	10
MYH13	7.46E+07	6.65E+07	11	1.07E+06	1.27E+06	10
LCP1	1.48E+08	1.47E+08	11	3.26E+05	4.06E+05	10
GDI1	7.66E+07	4.81E+07	11	2.14E+06	2.71E+06	10
GPI	7.36E+07	4.76E+07	11	1.55E+06	1.94E+06	10
PROS1	5.09E+07	3.46E+07	11	1.02E+05	2.15E+05	10
EIF3A	7.30E+07	3.91E+07	11	2.56E+05	3.43E+05	10
DKFZp459D1928; HEL-S-72p; Hspa8; HSPA8; LOC111153698; LOC117800504	9.44E+07	6.34E+07	11	5.41E+05	3.56E+05	10
NTM	7.72E+07	3.31E+07	11	7.16E+06	1.07E+07	10
NPC2	5.89E+07	2.49E+07	11	5.25E+06	4.11E+06	10
IGHV3-9	7.84E+07	4.33E+07	11	3.83E+06	7.45E+06	9
ADD1	1.21E+08	1.44E+08	11	4.45E+05	7.81E+05	10
ATP2B3	6.76E+07	4.25E+07	11	7.66E+04	9.03E+04	10
ANK1	1.79E+08	2.17E+08	11	8.05E+06	8.29E+06	10
UBA1	6.53E+07	3.80E+07	11	3.51E+05	4.02E+05	10
EFEMP1	6.00E+07	2.80E+07	11	1.60E+05	1.29E+05	10
CLSTN1	6.11E+07	3.90E+07	11	1.71E+05	1.88E+05	10
FLNC	7.66E+07	5.89E+07	11	7.86E+05	1.28E+06	10
FBLN1	4.98E+07	2.04E+07	11	1.86E+05	2.21E+05	10
TLN2	8.90E+07	1.27E+08	11	9.26E+05	8.43E+05	10
TLN1	6.50E+07	3.86E+07	11	3.73E+05	2.44E+05	10
GSTM3	7.06E+07	3.37E+07	11	9.03E+05	8.50E+05	10
ARF5	1.49E+08	1.61E+08	11	1.06E+05	1.81E+05	9
SPTB	6.49E+07	2.73E+07	11	5.96E+05	5.20E+05	10
PVALB	8.65E+07	6.32E+07	11	5.70E+06	9.48E+06	10
LRP1	4.72E+07	3.10E+07	11	2.45E+05	1.96E+05	10
PSAP	6.73E+07	4.51E+07	11	3.48E+06	3.16E+06	10
HSP90AA1	9.87E+07	8.88E+07	11	2.22E+05	1.72E+05	10
LOC101351914; LOC111146608; LOC113252869; YWHAZ	8.31E+07	6.02E+07	11	6.14E+05	4.17E+05	10
RBP4	7.54E+07	8.93E+07	11	3.21E+05	3.20E+05	10
TUBB2A	8.31E+07	5.71E+07	11	4.18E+05	5.38E+05	10
IGLC3	1.07E+08	1.15E+08	11	3.26E+06	2.52E+06	10
ECH1	6.68E+07	2.66E+07	11	6.85E+05	5.34E+05	10
AFM	5.20E+07	2.90E+07	11	3.12E+05	4.21E+05	10
LANCL1	6.38E+07	2.00E+07	11	2.26E+05	4.52E+05	10
PFN1	6.44E+07	2.98E+07	11	2.88E+06	3.13E+06	10
MIF	8.36E+07	7.62E+07	11	4.26E+06	5.60E+06	10
FKBP4	6.17E+07	3.12E+07	11	1.06E+06	3.02E+06	10
NEO1	7.37E+07	5.71E+07	11	9.49E+05	1.99E+06	10
FLNA	7.50E+07	4.69E+07	11	3.64E+05	3.15E+05	10
SEMA7A	5.91E+07	2.72E+07	11	1.53E+05	1.20E+05	10
LUM	1.04E+08	1.14E+08	11	6.14E+04	7.96E+04	10
PGM1	6.05E+07	4.41E+07	11	9.46E+05	7.86E+05	10
ACOT7	7.14E+07	4.11E+07	11	1.78E+05	2.02E+05	10
HDGF	9.13E+07	9.70E+07	11	8.49E+05	9.35E+05	9
PTPRN2	6.85E+07	5.17E+07	11	1.47E+05	2.06E+05	9
ALDH9A1	6.58E+07	4.06E+07	11	1.49E+05	1.06E+05	10
HSPA4L	5.84E+07	3.10E+07	11	4.16E+05	3.46E+05	10
GSS	5.33E+07	2.63E+07	11	3.62E+05	6.51E+05	10
VTN	5.28E+07	4.26E+07	11	6.37E+05	1.04E+06	10
EPRS1	4.99E+07	2.52E+07	11	1.23E+06	3.37E+06	10
EEA1	4.88E+07	2.62E+07	11	4.24E+06	9.00E+06	10
CAND1; LOC111142147	7.32E+07	3.70E+07	11	3.33E+06	4.68E+06	10
SIRPA	6.84E+07	4.95E+07	11	1.71E+06	1.71E+06	10
TAGLN	8.45E+07	1.16E+08	11	5.06E+04	6.63E+04	10
LAMC1	6.12E+07	5.35E+07	11	4.00E+05	3.67E+05	10
ATP1A1	7.67E+07	5.82E+07	11	2.06E+05	3.65E+05	10
FLNB	6.13E+07	4.66E+07	11	1.56E+06	2.91E+06	10
FABP4	4.99E+07	3.51E+07	11	2.07E+06	3.80E+06	10
FUCA2	6.18E+07	3.97E+07	11	2.04E+05	2.41E+05	10
NRXN3	5.15E+07	4.51E+07	11	5.30E+05	1.00E+06	10
ACTN4	5.16E+07	2.41E+07	11	1.68E+05	1.51E+05	10
CFL1	5.37E+07	3.39E+07	11	2.58E+06	4.97E+06	10
C4BPA	8.16E+07	5.26E+07	11	2.54E+05	3.33E+05	10
DBI	7.07E+07	5.87E+07	11	6.10E+06	5.95E+06	10
PSMA4	6.53E+07	3.84E+07	11	4.28E+04	2.59E+04	10
DDAH1	5.99E+07	4.98E+07	11	4.01E+06	4.04E+06	10
LOC100065732; LOC100155138; LOC100567848; LOC100587604; LOC101366680; LOC102739419; LOC102914778; LOC103669231; LOC110083393; LOC110143737; LOC110215276; LOC110296015; LOC111142377; LOC111162959; LOC112129091; LOC112812472; LOC113245958; LOC113907218; LOC114509275; LOC114597763; LOC116420883; LOC117678417; LOC118356237; LOC707215; TUBA2; Tuba3a; Tuba3b; TUBA3B; TUBA3C; TUBA3D; TUBA3E	8.12E+07	7.41E+07	11	1.57E+06	3.44E+06	10
KLK6	5.39E+07	2.55E+07	11	3.85E+06	4.69E+06	10
LAP3	5.90E+07	4.96E+07	11	5.37E+05	7.37E+05	10
KRT9	9.44E+07	1.28E+08	11	1.81E+07	2.09E+07	10
GSTM2	6.27E+07	4.95E+07	11	1.20E+06	1.14E+06	10
CSTB	4.97E+07	2.65E+07	11	2.57E+06	2.82E+06	10
F5	1.17E+08	1.26E+08	11	6.48E+05	7.02E+05	10
DDX17	4.29E+07	3.05E+07	11	2.33E+05	4.22E+05	10
CLEC3B	4.75E+07	3.17E+07	11	5.09E+05	8.60E+05	9
KRT2	5.05E+07	1.46E+07	11	8.90E+06	1.38E+07	10
NAP1L4	4.63E+07	2.80E+07	11	1.56E+06	2.24E+06	10
PCYT2	5.31E+07	2.86E+07	11	2.50E+05	5.17E+05	10
ENPP2	4.09E+07	1.92E+07	11	3.22E+05	3.04E+05	10
FABP7	6.62E+07	5.66E+07	11	1.95E+06	1.94E+06	10
ALDH1A1	7.50E+07	6.73E+07	11	3.22E+05	2.70E+05	10
AARS1	1.83E+08	2.16E+08	11	2.98E+06	2.53E+06	10
IGLV3-9	7.21E+07	6.56E+07	11	1.54E+05	1.88E+05	9
ILK	4.30E+07	2.57E+07	11	9.85E+04	1.54E+05	10
ITIH1	5.09E+07	3.01E+07	11	1.66E+05	1.34E+05	10
TPM3	5.03E+07	4.14E+07	11	3.42E+04	2.79E+04	10
LZTFL1	5.42E+07	4.04E+07	11	6.81E+04	8.97E+04	10
KIT	8.20E+07	7.83E+07	11	6.63E+05	6.16E+05	10
YWHAQ	1.83E+08	2.19E+08	11	1.02E+06	2.09E+06	10
FAM3C	4.12E+07	1.95E+07	11	1.98E+06	1.84E+06	10
VCL	5.92E+07	4.48E+07	11	3.08E+05	2.35E+05	10
SPTA1	6.17E+07	3.59E+07	11	4.16E+05	7.01E+05	10
FABP5	6.35E+07	4.91E+07	11	4.17E+06	4.38E+06	10
APOA2	5.09E+07	3.72E+07	11	1.90E+05	1.91E+05	10
SERPINF2	4.19E+07	1.77E+07	11	9.45E+04	1.26E+05	10
ANXA6	4.52E+07	2.37E+07	11	7.23E+05	6.93E+05	10
ACTBL2	5.00E+07	5.08E+07	11	9.14E+04	1.13E+05	10
CTSD	7.70E+07	1.17E+08	11	3.42E+05	2.30E+05	10
QSOX1	3.99E+07	2.03E+07	11	1.38E+05	1.48E+05	10
RNASE1	3.88E+07	2.69E+07	11	6.07E+06	5.92E+06	10
GLO1	4.99E+07	4.00E+07	11	3.63E+05	5.14E+05	10
APLP2	3.64E+07	1.45E+07	11	4.46E+05	4.74E+05	10
RELN	3.06E+07	1.50E+07	11	1.16E+06	1.35E+06	10
MAP2	7.95E+07	7.27E+07	11	1.18E+06	1.42E+06	10
PGD	3.98E+07	2.00E+07	11	1.05E+06	2.04E+06	10
ASS1	4.01E+07	2.10E+07	11	2.78E+05	2.65E+05	10
AHCYL2	4.00E+07	2.50E+07	11	1.21E+05	1.87E+05	10
CACNA2D1	4.86E+07	4.39E+07	11	2.38E+05	1.35E+05	10
COL3A1	5.07E+07	6.21E+07	11	2.20E+05	2.90E+05	10
LGALS1	5.31E+07	4.28E+07	11	5.56E+05	6.21E+05	10
THBS4	6.22E+07	7.57E+07	11	3.39E+05	3.39E+05	10
AMBP	5.92E+07	5.41E+07	11	3.56E+05	5.33E+05	10
FASN	3.60E+07	1.96E+07	11	7.63E+05	1.22E+06	10
AMPD2	3.71E+07	1.94E+07	11	8.43E+04	9.03E+04	10
GM2A	3.36E+07	1.46E+07	11	1.66E+05	1.95E+05	10
PLXNB2	4.64E+07	3.86E+07	11	9.93E+05	2.77E+06	10
AKR1B1	3.80E+07	2.61E+07	11	2.07E+06	1.93E+06	10
HUWE1	5.91E+07	5.50E+07	11	2.74E+06	2.56E+06	10
TPM1	5.04E+07	4.17E+07	11	2.52E+04	2.57E+04	8
ADH5	4.34E+07	3.09E+07	11	4.37E+05	6.43E+05	10
COL1A2	1.69E+08	4.26E+08	11	1.14E+06	2.03E+06	10
LTBP2	3.39E+07	2.51E+07	11	5.40E+05	9.06E+05	10
CTTN	4.34E+07	2.78E+07	11	5.01E+05	8.93E+05	10
CALD1	4.76E+07	4.74E+07	11	1.22E+06	2.13E+06	10
FAT2	3.32E+07	1.79E+07	11	7.27E+05	1.04E+06	10
LSAMP	2.95E+07	1.38E+07	11	2.38E+05	2.25E+05	10
KLC1	4.81E+07	2.87E+07	11	1.57E+06	2.28E+06	10
BCAN	2.80E+07	1.25E+07	11	1.15E+06	1.13E+06	10
GDI2	3.69E+07	2.22E+07	11	1.30E+05	1.58E+05	10
CPS1	4.02E+07	4.70E+07	11	3.28E+05	3.97E+05	10
SCG3	3.19E+07	1.86E+07	11	4.17E+05	4.14E+05	10
NELL2	3.80E+07	3.00E+07	11	5.70E+05	1.45E+06	10
VWF	4.54E+07	3.75E+07	11	8.56E+05	1.23E+06	10
USP14	2.31E+07	1.71E+07	11	1.47E+05	1.77E+05	10
HSPG2	4.70E+07	6.71E+07	11	9.05E+05	8.80E+05	10
PPM1A	3.08E+07	3.27E+07	11	7.08E+05	8.84E+05	10
EEF1A1P5	5.25E+07	4.18E+07	11	1.18E+06	2.21E+06	10
PTPRM	3.69E+07	3.16E+07	11	2.54E+06	5.00E+06	10
PRDX5	6.93E+07	8.50E+07	11	7.22E+05	5.18E+05	10
CTH	3.14E+07	1.08E+07	11	3.00E+05	2.63E+05	8
IGFBP2	2.84E+07	1.55E+07	11	1.37E+05	1.23E+05	10
PTPN11	3.73E+07	2.80E+07	11	1.98E+05	1.93E+05	10
ATP2B2	3.82E+07	2.38E+07	11	1.41E+05	2.29E+05	10
ACY1; HEL-S-5	5.08E+07	5.11E+07	11	5.29E+04	6.09E+04	8
PTPRZ1	3.17E+07	1.99E+07	11	8.77E+04	7.18E+04	10
ACTR3	4.85E+07	4.57E+07	11	1.91E+04	1.69E+04	10
TNC	3.23E+07	3.25E+07	11	6.66E+04	6.20E+04	10
GOT2	3.50E+07	2.32E+07	11	2.41E+05	2.63E+05	10
CPE	6.72E+07	1.43E+08	11	1.52E+05	1.77E+05	10
VCAN	3.84E+07	3.14E+07	11	1.21E+05	2.04E+05	10
GPD1L	2.80E+07	1.33E+07	11	2.36E+05	1.83E+05	10
MARCKS	3.04E+07	1.81E+07	11	8.39E+05	9.07E+05	10
YWHAB	4.05E+07	2.21E+07	11	1.30E+06	8.30E+05	10
EEF2	3.29E+07	1.85E+07	11	1.78E+06	2.55E+06	10
TALDO1	4.40E+07	2.96E+07	11	2.11E+05	1.96E+05	10
PHGDH	3.38E+07	2.02E+07	11	1.05E+06	1.49E+06	10
YWHAG	3.44E+07	2.48E+07	11	1.25E+05	1.80E+05	10
COL6A2	3.05E+07	2.21E+07	11	2.81E+05	3.40E+05	10
LAMA2	4.08E+07	6.01E+07	11	3.24E+05	3.23E+05	10
HSPA5	3.16E+07	1.66E+07	11	4.77E+05	4.62E+05	10
SERPINB1	3.00E+08	4.22E+08	11	2.20E+05	3.08E+05	10
HSPA1A	3.55E+07	2.85E+07	11	4.40E+05	2.84E+05	10
PCMT1	4.43E+07	3.83E+07	11	3.92E+05	2.75E+05	9
SEC31A	2.67E+07	1.04E+07	11	4.58E+05	1.10E+06	10
MAP1B	3.47E+07	3.22E+07	11	1.47E+06	1.48E+06	10
CD14	2.96E+07	2.03E+07	11	7.33E+04	5.85E+04	10
VCP	4.11E+07	2.23E+07	11	7.92E+05	1.81E+06	10
CAT	2.86E+07	1.48E+07	11	2.61E+05	2.82E+05	10
ANK2	2.82E+07	1.58E+07	11	1.95E+06	2.61E+06	10
MAP4	2.74E+07	1.63E+07	11	4.77E+05	7.53E+05	10
PZP	2.89E+07	1.94E+07	11	1.13E+05	1.70E+05	10
OGN	3.18E+07	1.86E+07	11	6.93E+05	6.69E+05	10
AHCY	2.74E+07	1.27E+07	11	5.83E+04	5.61E+04	10
TGFBI	8.90E+07	1.14E+08	11	2.99E+05	5.83E+05	10
MAP1A	5.32E+07	5.92E+07	11	5.71E+05	5.40E+05	10
PSMD3	4.58E+07	4.55E+07	11	4.47E+04	6.75E+04	10
NRXN1	6.31E+07	7.32E+07	11	3.74E+05	3.14E+05	10
GPD1	3.13E+07	2.16E+07	11	2.37E+05	2.63E+05	10
EZR	3.93E+07	3.04E+07	11	1.39E+07	1.88E+07	10
ST13	2.38E+07	2.33E+07	11	3.10E+05	2.45E+05	10
AK1	4.61E+07	4.88E+07	11	1.58E+06	1.82E+06	10
STIP1	2.66E+07	1.24E+07	11	1.87E+05	1.80E+05	10
Ap2b1; AP2B1; LOC101351101; LOC111151383	3.38E+07	3.22E+07	11	1.28E+05	1.93E+05	10
G6PD	3.09E+07	1.40E+07	11	1.56E+05	1.39E+05	10
LTBP4	3.13E+07	3.20E+07	11	1.40E+05	2.00E+05	10
CNDP2	3.12E+07	2.15E+07	11	9.24E+04	8.13E+04	10
KIF5B	1.93E+07	9.67E+06	11	3.26E+05	3.93E+05	10
VAT1L	2.80E+07	1.16E+07	11	7.77E+07	2.45E+08	10
LMNA	2.53E+07	1.61E+07	11	1.54E+05	1.33E+05	10
STXBP1	4.47E+07	4.42E+07	11	2.71E+05	5.03E+05	10
IGHV3-43	3.26E+07	3.18E+07	11	1.13E+05	1.15E+05	10
PYGL	2.45E+07	7.83E+06	11	1.12E+06	1.93E+06	10
ITIH3	2.75E+07	2.52E+07	11	1.21E+05	1.33E+05	10
TBCA	2.65E+07	9.01E+06	11	5.17E+05	5.70E+05	10
NFASC	2.69E+07	1.89E+07	11	2.88E+05	2.46E+05	10
SORBS1	1.10E+08	1.83E+08	11	2.07E+05	3.50E+05	10
SLC9A3R1	3.10E+07	2.48E+07	11	2.00E+05	1.59E+05	10
AQP4	2.31E+07	1.58E+07	11	2.37E+06	2.94E+06	10
CD163	2.94E+07	1.85E+07	11	2.75E+05	1.90E+05	10
ISYNA1	2.99E+07	1.83E+07	11	3.01E+05	3.90E+05	10
DPYSL3	3.19E+07	2.47E+07	11	2.81E+05	2.68E+05	10
JCHAIN	5.38E+07	5.97E+07	11	1.58E+05	1.78E+05	10
IGFBP7	2.56E+07	1.60E+07	11	7.00E+05	7.73E+05	10
IGFBP3	3.60E+07	3.06E+07	11	2.74E+04	2.68E+04	10
HEL-S-303; LOC101356254; LOC111153892; PA1B2; Pafah1b2; PAFAH1B2; PAFAH1B2P68402; SIDT2	2.42E+07	1.49E+07	11	3.79E+04	2.98E+04	10
KLKB1	2.47E+07	1.37E+07	11	4.42E+04	3.76E+04	10
SAA2	4.95E+07	5.26E+07	11	6.83E+05	6.30E+05	10
ALDH4A1	2.64E+07	1.81E+07	11	4.32E+04	4.07E+04	10
CLIC1	2.74E+07	2.01E+07	11	4.47E+05	6.09E+05	10
MYH6	7.35E+07	1.42E+08	11	6.26E+05	6.89E+05	10
TPPP3	2.64E+07	2.00E+07	11	4.23E+05	4.14E+05	10
ACTA1	3.36E+07	3.51E+07	11	2.04E+04	2.17E+04	10
PAFAH1B1	3.10E+07	1.74E+07	11	3.27E+05	4.84E+05	10
UBE2L3	2.65E+07	2.52E+07	11	1.82E+05	2.98E+05	10
ANXA5	3.33E+07	2.38E+07	11	7.36E+04	4.33E+04	10
CNTN2	1.84E+07	9.19E+06	11	2.82E+05	2.32E+05	10
COPA	6.82E+07	1.65E+08	11	1.07E+06	1.31E+06	10
TIMP1	2.17E+07	1.33E+07	11	4.34E+04	2.85E+04	10
AKR7A2	6.14E+07	7.12E+07	11	1.93E+05	2.21E+05	10
TARS1	1.20E+08	1.85E+08	11	1.77E+06	4.94E+06	10
MYOF	3.21E+07	2.99E+07	11	2.22E+05	2.76E+05	10
PDLIM1	3.02E+07	2.61E+07	11	3.49E+04	5.21E+04	8
IGSF8	2.72E+07	2.43E+07	11	2.36E+05	1.97E+05	10
AHCYL1	4.45E+07	5.13E+07	11	4.08E+05	6.27E+05	9
IGHV3-7	4.93E+07	6.35E+07	11	1.94E+05	1.91E+05	9
LYZ	3.45E+07	3.67E+07	11	1.12E+06	1.46E+06	10
CAST	1.90E+07	1.29E+07	11	9.06E+04	8.57E+04	9
KHSRP	6.33E+07	8.62E+07	11	6.20E+05	7.68E+05	10
SOD3	1.84E+07	1.17E+07	11	1.89E+05	4.67E+05	9
CUL5	2.64E+07	2.79E+07	11	7.85E+04	1.17E+05	10
LCAT	2.95E+07	2.22E+07	11	6.17E+05	1.23E+06	10
CSRP1	2.16E+07	1.69E+07	11	1.69E+05	2.86E+05	9
MAPRE1	2.63E+07	1.22E+07	11	3.54E+06	3.00E+06	10
AKAP12	2.26E+07	1.39E+07	11	5.77E+05	4.98E+05	10
NCAN	1.90E+07	7.67E+06	11	5.71E+05	1.51E+06	10
DCTN1	1.76E+07	8.36E+06	11	2.87E+05	2.94E+05	10
ACTN2	6.12E+07	8.65E+07	11	4.52E+05	4.10E+05	10
SH3BGRL	3.18E+07	3.14E+07	11	1.02E+06	1.23E+06	10
SLC12A2	1.78E+07	1.16E+07	11	3.42E+04	3.60E+04	10
CALB2	2.69E+07	2.21E+07	11	3.88E+05	6.62E+05	9
NCAM2	2.44E+07	2.15E+07	11	2.15E+05	1.45E+05	10
SPAG9	2.86E+07	2.07E+07	11	2.43E+05	3.15E+05	9
GLOD4	2.25E+07	1.17E+07	11	4.87E+05	6.86E+05	10
LGALS3BP	1.82E+07	1.05E+07	11	1.55E+05	2.66E+05	10
NME2	3.01E+07	2.12E+07	11	8.97E+04	7.87E+04	10
PEBP4	4.01E+07	4.19E+07	11	2.83E+05	2.68E+05	10
DSG2	2.79E+07	2.89E+07	11	1.29E+05	2.32E+05	10
FHL1	2.49E+07	1.63E+07	11	3.01E+05	3.57E+05	10
F10	2.05E+07	1.22E+07	11	1.00E+05	1.47E+05	9
PEA15	2.00E+07	7.58E+06	11	2.69E+05	2.91E+05	10
KRT16	3.34E+07	3.38E+07	11	2.44E+06	3.90E+06	10
EPB41L2	2.11E+07	9.75E+06	11	3.34E+05	4.74E+05	10
ECM1	1.71E+07	8.65E+06	11	9.12E+04	7.17E+04	10
SCRN1	2.25E+07	1.43E+07	11	4.91E+05	9.23E+05	10
SEPTIN7	2.26E+07	1.37E+07	11	4.27E+05	8.17E+05	10
MAPK1	1.80E+07	7.49E+06	11	1.48E+05	2.17E+05	10
ESD	2.49E+07	1.74E+07	11	2.26E+05	2.98E+05	10
COL14A1	7.94E+07	1.09E+08	11	4.42E+05	4.97E+05	10
WDR1	2.38E+07	1.60E+07	11	2.77E+05	2.58E+05	10
PAPLN	2.26E+07	1.80E+07	11	7.47E+04	1.38E+05	10
ADK	3.14E+07	2.72E+07	11	2.63E+05	3.12E+05	10
VCAM1	3.60E+07	6.13E+07	11	5.22E+05	7.57E+05	10
TAGLN2	1.75E+07	7.36E+06	11	3.37E+05	4.70E+05	10
HSPA9	2.51E+07	2.57E+07	11	1.17E+05	2.44E+05	10
ITIH5	5.25E+07	6.25E+07	11	4.77E+04	5.78E+04	10
MASP1	1.79E+07	7.58E+06	11	7.56E+05	1.01E+06	10
BLVRB	2.57E+07	1.35E+07	11	1.13E+06	8.76E+05	10
PGLYRP2	1.84E+07	9.93E+06	11	1.32E+05	1.42E+05	10
CRYL1	2.38E+07	1.30E+07	11	2.74E+06	4.05E+06	10
MAT2B	3.14E+07	3.51E+07	11	6.50E+04	9.64E+04	10
TYMP	3.38E+07	3.06E+07	11	1.00E+05	1.27E+05	10
LMNB2	1.94E+07	6.26E+06	11	2.57E+05	2.35E+05	8
NPTX1	1.48E+07	6.91E+06	11	1.75E+05	1.93E+05	10
CYFIP1	1.59E+07	7.87E+06	11	1.66E+05	3.15E+05	10
CD59	1.94E+07	8.06E+06	11	2.85E+06	2.61E+06	10
LRBA	2.84E+07	3.00E+07	11	8.48E+05	1.27E+06	10
LTA4H	2.09E+07	1.07E+07	11	2.61E+05	2.72E+05	10
CALM2	2.57E+07	2.05E+07	11	7.13E+05	7.42E+05	10
MRC1	2.20E+07	7.42E+06	11	1.15E+06	9.71E+05	10
CDH2	1.77E+07	8.98E+06	11	9.49E+04	1.24E+05	10
PPIB	2.22E+07	1.89E+07	11	1.62E+05	2.40E+05	10
CDH13	1.72E+07	8.54E+06	11	2.41E+05	3.42E+05	10
HEL-S-276; PSMA7	4.08E+07	6.41E+07	11	3.94E+05	8.70E+05	9
C16orf89	1.61E+07	1.32E+07	11	7.29E+04	8.25E+04	6
RGMA	2.62E+07	1.90E+07	11	1.72E+04	2.79E+04	7
COL6A1	1.61E+07	6.57E+06	11	1.38E+06	2.31E+06	10
	1.66E+07	8.23E+06	11	1.63E+05	1.95E+05	10
VARS1	3.49E+07	3.23E+07	11	2.60E+05	3.28E+05	10
UGP2	1.72E+07	7.75E+06	11	1.12E+05	2.12E+05	10
AKR1A1	2.71E+07	2.13E+07	11	1.25E+06	1.35E+06	10
HNRNPA1	1.80E+07	6.91E+06	11	1.78E+05	3.18E+05	10
COL28A1	8.58E+07	2.14E+08	11	1.34E+05	1.18E+05	10
MYH14	1.63E+07	9.03E+06	11	5.18E+05	5.72E+05	10
MAP6	3.96E+07	4.65E+07	11	1.29E+05	1.98E+05	10
MANBA	2.78E+07	2.44E+07	11	2.64E+04	3.44E+04	9
BTD	1.95E+07	1.13E+07	11	5.10E+04	3.40E+04	10
ROBO1	1.60E+07	1.00E+07	11	1.18E+05	1.33E+05	10
FGFR1	1.57E+07	1.33E+07	11	7.06E+04	8.92E+04	8
SEMA6D	1.48E+07	7.39E+06	11	6.04E+04	7.41E+04	10
SND1	2.64E+07	2.37E+07	11	1.54E+05	2.34E+05	10
HSP90B1	1.48E+07	9.92E+06	11	9.63E+05	1.69E+06	10
RAB1A	1.95E+07	9.03E+06	11	7.13E+04	8.38E+04	10
CCT2; HEL-S-100n	2.91E+07	2.20E+07	11	1.32E+05	2.09E+05	9
ANXA1	2.67E+07	2.25E+07	11	2.89E+05	4.87E+05	10
PCSK1N	1.37E+07	6.51E+06	11	1.97E+05	2.00E+05	10
ACO1	2.04E+07	1.23E+07	11	1.27E+05	1.11E+05	10
PRKCA	2.08E+07	1.30E+07	11	5.24E+04	1.11E+05	9
APCS	1.97E+07	1.31E+07	11	2.67E+05	5.91E+05	9
HRG	1.92E+07	1.18E+07	11	3.54E+04	3.23E+04	10
HNRNPUL2	1.40E+07	1.28E+07	11	1.58E+05	3.01E+05	10
	1.84E+07	1.45E+07	11	5.75E+04	5.11E+04	10
THBS1	1.86E+07	1.20E+07	11	5.90E+05	1.37E+06	9
SERPINA7	1.41E+07	9.72E+06	11	2.65E+05	2.61E+05	10
RDX	1.81E+07	1.28E+07	11	2.94E+04	2.08E+04	10
PLIN3	1.39E+07	9.42E+06	11	1.61E+05	2.56E+05	10
ATP6V1A	2.53E+07	2.36E+07	11	6.35E+05	7.37E+05	10
SHTN1	1.70E+07	1.09E+07	11	4.52E+04	4.78E+04	10
F13A1	2.38E+07	2.94E+07	11	1.45E+05	1.44E+05	10
SAA1	4.03E+07	5.09E+07	11	8.93E+04	1.04E+05	10
CAPN1	1.71E+07	1.15E+07	11	1.87E+05	1.68E+05	10
ALDH7A1	1.77E+07	1.13E+07	11	1.92E+05	2.85E+05	10
MAP4	1.20E+07	8.18E+06	11	4.02E+05	3.33E+05	10
RTCB	1.42E+07	1.04E+07	11	9.10E+04	1.53E+05	10
CMBL	1.76E+07	1.12E+07	11	7.86E+05	9.08E+05	10
ACO2	6.15E+07	8.03E+07	11	1.89E+05	2.74E+05	10
PDCD6IP	1.61E+07	6.90E+06	11	5.21E+04	3.17E+04	9
EIF4G1	1.65E+07	1.48E+07	11	5.96E+06	1.78E+07	10
IDH1	2.31E+07	1.87E+07	11	1.58E+05	1.53E+05	10
TPP2	1.53E+07	7.72E+06	11	1.42E+06	1.41E+06	10
RTN4	1.51E+07	9.57E+06	11	2.36E+05	2.33E+05	10
MYH8	1.33E+07	8.93E+06	11	3.97E+05	5.29E+05	9
OTC	1.92E+07	1.35E+07	11	2.03E+04	1.50E+04	8
RAN	1.68E+07	9.14E+06	11	5.56E+05	6.77E+05	10
DNM1	2.22E+07	2.18E+07	11	5.34E+04	5.18E+04	10
CDH11	1.77E+07	1.07E+07	11	1.98E+05	3.24E+05	10
SLK	2.54E+07	2.02E+07	11	4.25E+05	7.44E+05	10
SERPINA4	1.38E+07	5.45E+06	11	1.28E+05	2.09E+05	10
SCIN	1.85E+07	1.41E+07	11	2.58E+05	2.14E+05	10
IGHV3-74	3.66E+07	4.30E+07	11	2.78E+05	3.18E+05	10
AHSA1	3.17E+07	3.77E+07	11	6.97E+04	7.42E+04	10
IGFBP6	1.77E+07	1.72E+07	11	1.25E+05	1.42E+05	10
RNH1	1.95E+07	1.51E+07	11	7.79E+04	7.97E+04	10
ALDH2	2.28E+07	2.68E+07	11	1.02E+05	1.73E+05	10
CSPG4	2.44E+07	4.03E+07	11	3.10E+05	3.18E+05	10
PRNP	1.37E+07	6.66E+06	11	1.63E+06	1.82E+06	10
ALDH5A1	1.42E+07	1.75E+07	11	5.39E+04	4.00E+04	10
TUBA4A	2.20E+07	1.75E+07	11	1.19E+05	1.06E+05	10
TXN	7.40E+07	1.77E+08	11	1.40E+06	1.36E+06	10
NIT2	4.31E+07	1.08E+08	11	4.98E+05	8.01E+05	10
AK2	1.41E+07	1.21E+07	11	7.37E+04	9.26E+04	9
PPBP	1.57E+07	1.22E+07	11	1.97E+05	2.57E+05	9
GSR	1.80E+07	1.17E+07	11	5.46E+04	7.49E+04	10
CCT6A	2.11E+07	1.97E+07	11	2.08E+05	1.86E+05	10
HEXB	2.40E+07	1.61E+07	11	7.73E+05	1.29E+06	10
DNM1L	1.21E+07	4.15E+06	11	2.07E+05	2.49E+05	10
PCCB	1.45E+07	1.07E+07	11	6.34E+04	9.61E+04	10
CADM1	1.33E+07	1.05E+07	11	5.18E+04	6.27E+04	10
NAXE	1.64E+07	9.52E+06	11	8.46E+04	1.41E+05	10
LMAN2	1.66E+07	1.27E+07	11	9.51E+04	1.26E+05	10
PFKM	3.89E+07	9.42E+07	11	3.61E+05	6.89E+05	10
LTBP1	1.70E+07	1.19E+07	11	5.15E+04	4.78E+04	10
NPTXR	1.39E+07	4.27E+06	11	4.26E+05	3.94E+05	10
AKR1C3	1.99E+07	1.55E+07	11	1.92E+05	3.29E+05	10
PTPRG	1.51E+07	9.15E+06	11	9.45E+04	9.21E+04	10
PTK7	1.49E+07	8.23E+06	11	6.19E+05	1.27E+06	10
CCT4	1.72E+07	9.51E+06	11	2.51E+05	4.48E+05	9
EHD1	1.38E+07	7.56E+06	11	1.10E+05	1.11E+05	9
ATP5F1A; HEL-S-123m	1.48E+07	6.21E+06	11	8.72E+05	6.73E+05	10
RHOC	1.74E+07	1.00E+07	11	6.25E+04	6.63E+04	10
DAG1	1.40E+07	7.54E+06	11	8.34E+04	1.54E+05	10
PNP	1.60E+07	8.10E+06	11	1.99E+05	2.55E+05	10
IGHV3-30	3.05E+07	3.43E+07	11	3.65E+05	3.51E+05	10
HEL-S-1a; PDXK	1.51E+07	6.50E+06	11	9.27E+04	1.20E+05	10
AKR1C1	2.12E+07	1.74E+07	11	6.25E+04	1.07E+05	10
HPR	2.56E+07	2.37E+07	11	2.26E+06	4.92E+06	10
GH1	1.47E+07	8.05E+06	11	6.30E+04	6.24E+04	10
ALCAM	1.17E+07	2.32E+06	11	8.08E+04	4.28E+04	10
HK1	1.38E+07	7.00E+06	11	1.05E+06	2.05E+06	10
ENO3	1.97E+07	1.69E+07	11	1.45E+07	1.98E+07	10
NRXN2	1.15E+07	6.67E+06	11	2.68E+05	2.86E+05	10
MYH1	1.83E+07	2.46E+07	11	4.51E+05	5.36E+05	10
HSPA4	1.59E+07	1.18E+07	11	2.63E+05	3.11E+05	10
NUCB1	2.01E+07	1.62E+07	11	1.83E+07	2.34E+07	10
F12	1.25E+07	1.01E+07	11	5.76E+04	7.06E+04	10
P4HB	1.64E+07	1.35E+07	11	1.25E+05	1.64E+05	10
TJP1	1.69E+07	1.84E+07	11	9.84E+04	1.77E+05	10
DCLK1	1.28E+07	6.11E+06	11	8.26E+05	2.00E+06	9
SPARC	1.32E+07	7.88E+06	11	8.15E+04	6.52E+04	9
ST13P5	1.50E+07	1.12E+07	11	1.83E+05	1.30E+05	10
MPO	3.15E+07	3.17E+07	11	9.08E+04	9.65E+04	10
ATP5F1B	1.50E+07	5.22E+06	11	4.10E+04	6.04E+04	10
PMP2	1.79E+07	1.83E+07	11	3.88E+05	8.98E+05	10
NSFL1C	4.19E+07	9.60E+07	11	8.17E+04	5.91E+04	10
EEF1G	1.04E+07	6.15E+06	11	8.30E+04	1.55E+05	10
PYGM	1.42E+07	8.45E+06	11	6.59E+04	7.96E+04	10
GSTA1	2.09E+07	2.02E+07	11	1.67E+05	2.32E+05	10
CAPG	2.31E+07	2.01E+07	11	1.03E+05	1.45E+05	10
PYGB	1.72E+07	1.71E+07	11	2.42E+05	4.38E+05	10
VSTM2A	1.28E+07	7.21E+06	11	3.47E+05	3.48E+05	8
HINT1	1.54E+07	1.03E+07	11	1.49E+05	1.50E+05	10
SEPTIN8	1.71E+07	1.61E+07	11	2.57E+05	2.62E+05	10
PLS3	1.87E+07	1.49E+07	11	5.31E+04	5.19E+04	10
ADD3	1.32E+07	7.88E+06	11	2.41E+05	2.11E+05	10
GNAI2	9.65E+06	8.99E+06	11	1.27E+05	1.89E+05	6
TXNRD1	2.21E+07	3.23E+07	11	2.72E+05	4.71E+05	10
PKM	2.44E+07	2.85E+07	11	4.62E+04	7.45E+04	9
KRT5	2.59E+07	3.25E+07	11	1.55E+06	2.35E+06	10
ARPC2	2.36E+07	2.45E+07	11	1.05E+05	1.42E+05	10
MYH11	1.54E+07	1.01E+07	11	1.24E+05	1.62E+05	10
LASP1	1.69E+07	1.19E+07	11	2.68E+05	3.93E+05	10
FTH1	2.70E+07	2.94E+07	11	4.04E+04	3.81E+04	10
LAMB1	1.32E+07	5.19E+06	11	8.56E+04	9.04E+04	10
SYNJ1	1.28E+07	7.03E+06	11	6.66E+05	1.34E+06	10
TNR	1.55E+07	1.03E+07	11	3.87E+05	6.18E+05	10
HNRNPL	1.80E+07	3.46E+07	11	4.82E+04	5.07E+04	10
CSF1R	1.07E+07	1.18E+07	11	3.84E+04	3.37E+04	10
LPA	1.26E+07	6.35E+06	11	6.59E+05	1.42E+06	10
CUL3	1.30E+07	8.32E+06	11	4.65E+04	3.87E+04	9
MST1	2.00E+07	2.83E+07	11	1.05E+05	1.30E+05	10
CALR	1.20E+07	7.50E+06	11	2.38E+04	2.17E+04	10
PCDH1	1.11E+07	1.04E+07	11	9.63E+04	1.32E+05	9
HNRNPR	1.24E+07	1.07E+07	11	7.71E+04	1.01E+05	10
ANXA4	1.39E+07	9.26E+06	11	2.32E+05	4.36E+05	10
HSP90AB1	1.79E+07	1.63E+07	11	1.07E+06	1.44E+06	10
ATP2B4	1.17E+07	6.74E+06	11	6.22E+04	1.01E+05	10
THY1	1.13E+07	9.85E+06	11	1.05E+06	1.21E+06	9
MEGF8	1.14E+07	5.68E+06	11	1.85E+05	2.63E+05	10
CLIC4	1.09E+07	7.12E+06	11	1.43E+07	1.99E+07	10
YWHAH	1.65E+07	1.28E+07	11	1.40E+06	2.41E+06	10
HNRNPK	1.52E+07	1.01E+07	11	3.39E+05	4.28E+05	10
PTGR1	1.93E+07	1.76E+07	11	4.48E+04	5.05E+04	10
PTPRD	1.30E+07	1.04E+07	11	2.34E+05	3.80E+05	10
MPST	9.73E+06	5.77E+06	11	3.68E+05	3.42E+05	10
Pfn2; PFN2	1.56E+07	1.25E+07	11	2.30E+05	2.64E+05	9
CPN2	1.11E+07	6.38E+06	11	2.94E+05	2.92E+05	10
LBP	1.18E+07	1.17E+07	11	8.35E+05	1.66E+06	10
BASP1	1.19E+07	6.60E+06	11	4.39E+05	7.30E+05	10
TCP1	1.41E+07	1.28E+07	11	2.95E+05	3.67E+05	10
GAS6	1.19E+07	7.99E+06	11	8.91E+04	9.84E+04	9
ATP1A4	1.04E+07	9.61E+06	11	2.75E+04	3.03E+04	10
GNB2	1.15E+07	1.78E+07	11	8.42E+04	8.93E+04	9
TPD52L2	4.29E+07	6.86E+07	11	1.77E+05	2.53E+05	10
cRac1A; LOC107556616; LOC107698781; LOC111145636; LOC113047578; rac1; Rac1; RAC1; RAC2	1.36E+07	9.94E+06	11	5.96E+04	5.94E+04	9
LOC101347799; LOC111143461; Psma5; PSMA5	5.27E+07	1.42E+08	11	5.18E+04	4.85E+04	9
CNTNAP2	1.01E+07	5.50E+06	11	3.67E+04	2.99E+04	10
QDPR	1.17E+07	8.23E+06	11	1.55E+05	2.56E+05	10
CAPZB	1.36E+07	7.70E+06	11	4.97E+04	4.92E+04	10
BLVRA	1.56E+07	1.19E+07	11	1.10E+05	7.84E+04	10
PPP2R1A	9.40E+06	5.33E+06	11	2.36E+06	2.03E+06	10
UBE2V1	1.19E+07	7.24E+06	11	3.29E+05	3.47E+05	9
PFKL	8.13E+06	4.90E+06	11	6.56E+04	6.91E+04	8
MAPK3	1.26E+07	6.73E+06	11	3.72E+04	3.00E+04	10
NAMPT	1.50E+07	2.08E+07	11	4.95E+04	4.81E+04	9
ANXA7	1.05E+07	7.47E+06	11	6.18E+04	6.29E+04	10
EPHA4	9.88E+06	5.88E+06	11	2.58E+05	3.39E+05	10
PON1	1.26E+07	7.41E+06	11	3.24E+06	5.35E+06	10
PCOLCE	9.80E+06	6.08E+06	11	3.90E+04	6.20E+04	9
PLXDC2	1.06E+07	4.71E+06	11	9.08E+04	1.50E+05	10
KRT85	1.16E+07	6.98E+06	11	1.22E+06	2.19E+06	10
NDRG2	2.44E+07	3.83E+07	11	6.30E+06	1.22E+07	10
FH	1.22E+07	8.98E+06	11	7.57E+04	9.85E+04	10
DLG4	5.08E+07	8.85E+07	11	3.34E+04	2.69E+04	7
ARHGDIA; HEL-S-47e	1.08E+07	7.62E+06	11	1.80E+05	1.69E+05	10
AGL	1.37E+07	7.59E+06	11	1.13E+05	1.75E+05	10
SLC5A5	1.36E+07	1.21E+07	11	1.03E+06	1.37E+06	10
NUDC	1.06E+07	7.26E+06	11	8.20E+04	1.01E+05	10
FSCN1	1.43E+07	1.15E+07	11	5.42E+05	7.09E+05	10
ACLY	1.17E+07	5.45E+06	11	5.29E+04	5.00E+04	10
PGM2	1.22E+07	7.21E+06	11	6.23E+04	6.04E+04	10
GNB1	1.19E+07	1.09E+07	11	4.45E+04	3.86E+04	10
DECR1	9.69E+06	6.79E+06	11	5.35E+04	8.77E+04	8
PLCD1	1.73E+07	1.64E+07	11	6.07E+04	9.15E+04	10
PADI2	1.85E+07	2.15E+07	11	2.19E+04	2.82E+04	9
PREP	1.11E+07	6.83E+06	11	9.22E+04	1.10E+05	10
AP2M1	4.48E+07	1.13E+08	11	4.63E+04	6.23E+04	10
KBTBD11	8.83E+06	7.23E+06	11	5.19E+04	7.65E+04	10
GSTO1	1.42E+07	9.32E+06	11	2.26E+05	2.53E+05	10
ALDH6A1	1.09E+07	7.50E+06	11	2.11E+05	3.27E+05	10
HPD	1.18E+07	7.74E+06	11	3.90E+04	4.31E+04	10
MAN1A1	5.25E+07	8.06E+07	11	3.26E+04	3.04E+04	10
TMEM132A	1.21E+07	7.08E+06	11	6.25E+04	8.90E+04	10
CCT8	1.48E+07	1.75E+07	11	6.08E+04	7.79E+04	10
RNPEP	1.21E+07	7.91E+06	11	2.63E+06	4.03E+06	10
MYL1	1.81E+07	2.30E+07	11	1.26E+05	2.58E+05	10
SLC39A12	1.18E+07	5.31E+06	11	5.13E+04	7.39E+04	9
PTPRF	1.08E+07	8.36E+06	11	1.36E+05	9.12E+04	10
CRYZ	1.28E+07	9.45E+06	11	5.84E+04	6.79E+04	9
PAK2	2.08E+07	2.28E+07	11	4.30E+04	6.06E+04	10
TFG	1.04E+07	5.12E+06	11	6.85E+04	6.85E+04	8
KRT19	7.66E+06	3.46E+06	11	1.88E+04	2.36E+04	10
MTPN	1.05E+07	7.52E+06	11	2.31E+05	2.31E+05	10
HSPH1	8.96E+06	5.73E+06	11	3.07E+05	5.45E+05	10
MBP	1.21E+07	1.13E+07	11	9.52E+04	1.75E+05	10
LARS1	9.71E+06	7.29E+06	11	7.88E+04	8.15E+04	10
WFIKKN2	2.35E+07	2.79E+07	11	2.23E+04	2.09E+04	10
GPX4	1.07E+07	8.32E+06	11	1.13E+05	2.47E+05	10
TJP2	9.81E+06	7.38E+06	11	1.20E+05	1.09E+05	9
DDAH2	8.90E+06	5.39E+06	11	4.03E+05	7.04E+05	10
NUTF2	1.14E+07	7.81E+06	11	4.84E+05	8.91E+05	9
FARSB	1.25E+07	8.49E+06	11	5.25E+04	4.45E+04	9
SEPTIN10	2.70E+07	2.87E+07	11	3.78E+04	2.98E+04	10
IARS1	9.27E+06	5.58E+06	11	3.01E+04	2.92E+04	10
FABP3	1.65E+07	1.48E+07	11	2.30E+05	2.66E+05	10
CD109	9.36E+06	4.57E+06	11	1.64E+05	1.61E+05	10
GLUD1	1.12E+07	1.02E+07	11	5.09E+05	6.35E+05	10
SORL1	7.55E+06	3.62E+06	11	1.54E+05	2.35E+05	10
EPB41L1	7.67E+06	5.44E+06	11	2.85E+05	5.26E+05	10
SORD	1.21E+07	9.98E+06	11	7.18E+04	8.31E+04	10
CMPK1	1.39E+07	1.34E+07	11	3.35E+05	4.32E+05	10
CCT7	1.40E+07	8.38E+06	11	8.56E+04	7.75E+04	10
CNTNAP4	1.00E+07	3.53E+06	11	1.52E+06	3.36E+06	9
EPB41L3	8.15E+06	4.10E+06	11	1.21E+05	2.21E+05	10
CTSB	8.77E+06	5.18E+06	11	1.37E+05	1.60E+05	10
PPP1R1B	1.04E+07	1.11E+07	11	2.55E+05	3.22E+05	10
PACSIN1	3.62E+07	9.29E+07	11	3.53E+04	3.36E+04	10
PPA1	1.25E+07	1.11E+07	11	1.43E+05	2.04E+05	10
ATRN	8.67E+06	5.82E+06	11	1.31E+06	1.51E+06	10
TAGLN3	8.42E+06	4.22E+06	11	1.41E+05	1.04E+05	10
RUVBL1	1.23E+07	1.19E+07	11	3.64E+04	5.77E+04	10
CD5L	2.40E+07	3.03E+07	11	1.37E+05	2.42E+05	10
CACYBP	1.35E+07	9.88E+06	11	3.61E+04	4.84E+04	8
	1.31E+07	1.10E+07	11	1.34E+05	1.38E+05	10
ABHD14B	1.27E+07	9.52E+06	11	2.40E+05	3.74E+05	10
IGLV1-40	1.30E+07	1.02E+07	11	8.50E+04	1.90E+05	9
NARS1	1.14E+07	8.13E+06	11	6.36E+04	7.71E+04	10
SEC14L2	2.28E+07	4.88E+07	11	3.47E+04	3.69E+04	10
CAVIN1	6.17E+06	3.34E+06	11	3.49E+04	2.77E+04	9
IGKV1-6	1.10E+07	8.85E+06	11	4.69E+04	7.39E+04	9
EFHD2	8.56E+06	4.65E+06	11	4.70E+04	7.77E+04	9
EPHX2	1.21E+07	1.40E+07	11	9.48E+05	2.30E+06	10
ADGRL3	1.48E+07	1.55E+07	11	2.23E+05	3.62E+05	10
	1.31E+07	1.06E+07	11	2.38E+04	3.38E+04	5
APOL1	1.30E+07	1.35E+07	11	1.26E+04	9.02E+03	9
FERMT2	1.53E+07	1.28E+07	11	5.66E+04	6.04E+04	10
DPP3	8.40E+06	4.90E+06	11	1.76E+05	1.27E+05	10
MDH2	2.46E+07	2.88E+07	11	8.77E+04	8.23E+04	10
FKBP1A	1.53E+08	3.17E+08	11	1.06E+07	1.40E+07	10
FUBP1	1.82E+07	1.76E+07	11	9.56E+04	1.50E+05	10
TUBB6	1.37E+07	1.32E+07	11	5.18E+04	7.60E+04	8
HPRT1	9.47E+06	7.04E+06	11	4.05E+04	3.44E+04	9
ALDH3A1	7.15E+06	4.81E+06	11	7.67E+04	1.20E+05	8
PPM1B	7.48E+06	5.57E+06	11	4.20E+04	4.14E+04	10
SRI	1.05E+07	7.73E+06	11	1.01E+04	1.17E+04	8
C7orf24; GGCT	9.15E+06	4.28E+06	11	9.41E+05	1.81E+06	10
TPM1	1.17E+07	1.02E+07	11	7.29E+04	7.70E+04	10
NBL1	8.24E+06	4.67E+06	11	2.96E+04	4.06E+04	7
HSP90AA5P	7.50E+06	6.12E+06	11	4.99E+04	8.43E+04	8
RIDA	1.14E+07	1.38E+07	11	3.79E+04	4.69E+04	6
Dstn; DSTN; HEL32; LOC111141429; LOC112828084	9.69E+06	6.87E+06	11	1.44E+05	2.50E+05	10
MVP	1.10E+07	6.91E+06	11	6.09E+05	1.01E+06	10
CAPZB	1.47E+07	1.31E+07	11	3.88E+04	5.07E+04	5
GRIA4	7.39E+06	1.58E+06	11	1.04E+05	1.01E+05	10
GBE1	1.26E+07	9.45E+06	11	8.95E+04	1.64E+05	10
NIBAN2	2.08E+07	3.90E+07	11	8.85E+04	8.53E+04	10
ALAD	7.82E+06	3.87E+06	11	7.64E+04	1.19E+05	10
AHCYL2	8.57E+06	8.20E+06	11	1.91E+04	1.54E+04	3
KNG1	6.82E+06	4.36E+06	11	1.05E+05	1.25E+05	4
PPP1R7	1.29E+07	1.14E+07	11	3.89E+05	4.14E+05	10
IGFBP4	8.49E+06	5.63E+06	11	8.12E+04	8.04E+04	10
NEGR1	7.02E+06	2.88E+06	11	3.89E+04	4.38E+04	10
NANS	9.18E+06	6.30E+06	11	1.93E+06	4.05E+06	10
GLUD2	1.40E+07	2.31E+07	11	5.64E+04	9.95E+04	10
PSMD2	9.90E+06	6.48E+06	11	2.91E+05	6.68E+05	10
SEZ6L	7.49E+06	3.48E+06	11	5.81E+04	9.25E+04	10
SERPINA6	7.56E+06	5.76E+06	11	1.22E+05	1.70E+05	10
SPON1	6.90E+06	2.53E+06	11	1.04E+05	1.49E+05	10
MST1L	6.24E+06	3.15E+06	11	9.70E+05	8.67E+05	9
CCN3	2.53E+07	3.52E+07	11	7.00E+05	1.40E+06	10
HSPD1	1.28E+07	7.27E+06	11	1.33E+05	2.04E+05	10
CBR3	1.56E+07	2.09E+07	11	7.52E+04	8.46E+04	10
ATP2B2	6.00E+06	4.67E+06	11	1.17E+05	9.01E+04	8
UGDH	1.05E+08	1.97E+08	11	4.96E+05	4.91E+05	10
DPYSL5	1.00E+07	9.96E+06	11	5.01E+04	4.98E+04	9
MCAM	7.02E+06	2.77E+06	11	8.46E+04	8.10E+04	10
OTUB1	1.03E+07	4.45E+06	11	1.36E+05	2.35E+05	9
ATP1B1	1.62E+07	1.73E+07	11	3.39E+05	5.70E+05	10
ADAM22	8.74E+06	5.59E+06	11	4.95E+04	4.50E+04	10
CLTCL1	1.17E+07	1.26E+07	11	1.11E+05	1.77E+05	10
PCP4	7.18E+06	5.85E+06	11	1.06E+06	1.20E+06	9
PRELP	1.02E+07	1.01E+07	11	4.80E+04	2.57E+04	10
ASMTL	6.41E+06	2.32E+06	11	7.07E+04	8.83E+04	10
TIMP2	7.30E+06	3.44E+06	11	2.55E+04	2.48E+04	10
PENK	6.65E+06	2.84E+06	11	1.10E+06	1.11E+06	10
KRT8; LOC117800407	7.54E+06	4.54E+06	11	4.22E+04	7.53E+04	10
NQO1	1.77E+07	2.06E+07	11	2.34E+04	2.69E+04	9
PTPRS	8.64E+06	7.99E+06	11	8.56E+04	7.45E+04	10
CCT3	1.27E+07	1.86E+07	11	1.16E+05	1.59E+05	10
PGLS	8.62E+06	6.53E+06	11	8.04E+06	2.48E+07	10
MYH7	1.02E+07	6.17E+06	11	2.32E+06	5.52E+06	10
PSMA6	8.42E+06	5.49E+06	11	5.35E+04	5.33E+04	10
SNCG	9.59E+06	7.26E+06	11	2.37E+05	2.93E+05	10
COL18A1	7.68E+06	4.78E+06	11	8.98E+04	1.07E+05	10
CADM4	8.81E+06	8.15E+06	11	7.07E+04	1.22E+05	10
CHORDC1	6.27E+06	4.27E+06	11	4.65E+04	5.18E+04	10
USP5	6.56E+06	4.44E+06	11	8.37E+06	1.24E+07	9
CAMK2A	7.83E+06	5.69E+06	11	1.09E+05	1.31E+05	10
DDX1	1.15E+07	1.36E+07	11	9.27E+04	5.02E+04	10
LTF	8.17E+06	6.89E+06	11	1.28E+05	1.33E+05	10
CORO1B	9.99E+06	1.38E+07	11	2.78E+04	4.69E+04	8
ATP2B1	8.88E+06	9.42E+06	11	9.69E+04	1.43E+05	10
MAN2A2	7.46E+06	4.88E+06	11	1.39E+05	1.44E+05	10
SEPTIN2	7.66E+06	3.94E+06	11	7.01E+04	8.10E+04	10
L1CAM	7.00E+06	7.11E+06	11	6.52E+04	5.27E+04	10
CAPN2	9.05E+06	6.12E+06	11	5.59E+04	7.73E+04	9
PDIA3	7.81E+06	5.89E+06	11	1.03E+05	7.18E+04	10
TUBB	1.21E+07	1.19E+07	11	2.56E+04	1.82E+04	10
DDTL	9.27E+06	8.68E+06	11	2.74E+05	3.26E+05	10
FBP2	1.39E+07	1.36E+07	11	3.58E+05	6.94E+05	10
NCL	7.49E+06	4.76E+06	11	1.49E+05	1.99E+05	10
VPS35	8.16E+06	5.76E+06	11	2.73E+04	2.44E+04	10
PFKFB2	1.36E+07	2.14E+07	11	3.95E+04	3.79E+04	10
DBNL	7.51E+06	7.71E+06	11	6.42E+05	7.04E+05	10
PSME1	6.88E+06	4.58E+06	11	1.03E+05	1.02E+05	10
IPO5	3.55E+07	9.34E+07	11	2.55E+05	4.45E+05	10
CLSTN3	7.16E+06	4.63E+06	11	6.44E+04	5.41E+04	10
SH3GL2	1.07E+07	1.03E+07	11	2.67E+05	7.25E+05	10
PIGR	1.27E+07	1.86E+07	11	3.11E+05	4.46E+05	10
NID1	7.58E+06	7.52E+06	11	2.89E+04	2.07E+04	10
RARS1	6.28E+06	3.27E+06	11	3.93E+04	3.11E+04	10
DBN1	5.89E+06	3.40E+06	11	7.57E+04	1.11E+05	10
ATIC	8.64E+06	6.58E+06	11	6.72E+04	4.00E+04	10
COPS4	8.20E+06	9.44E+06	11	1.19E+05	3.17E+05	10
EEF1D	1.30E+07	2.06E+07	11	3.93E+04	7.06E+04	10
BHMT	9.59E+06	1.23E+07	11	6.56E+04	7.22E+04	10
CORO1A	7.34E+06	4.23E+06	11	1.93E+04	1.00E+04	9
SCRG1	8.46E+06	6.02E+06	11	4.93E+04	5.17E+04	9
SUSD5	1.17E+07	1.22E+07	11	7.08E+04	9.15E+04	10
HABP2	5.93E+06	2.65E+06	11	1.01E+05	1.21E+05	10
LHPP	1.06E+07	6.95E+06	11	3.67E+05	3.07E+05	10
SUGT1	5.27E+06	3.61E+06	11	3.30E+05	3.03E+05	10
PRPS1	1.28E+07	1.72E+07	11	1.22E+05	2.96E+05	9
DMGDH	8.26E+06	8.56E+06	11	3.35E+04	4.45E+04	10
FBLN5	8.84E+06	1.25E+07	11	1.57E+05	2.72E+05	9
PA2G4	9.06E+06	5.84E+06	11	1.76E+06	5.03E+06	10
SNX1	1.19E+07	1.21E+07	11	6.13E+04	8.25E+04	8
HBG1	6.86E+06	4.64E+06	11	3.96E+04	7.61E+04	9
FAH	8.29E+06	9.09E+06	11	7.56E+04	7.09E+04	9
APLP1	6.80E+06	3.76E+06	11	2.65E+05	2.73E+05	10
HAGH	7.00E+06	3.18E+06	11	7.07E+06	5.77E+06	10
DCN	9.26E+06	6.25E+06	11	4.51E+04	3.33E+04	10
RTN1	7.72E+06	7.11E+06	11	6.34E+04	1.37E+05	10
LOC101353736; LOC111158036; LOC112837724; Psmc5; PSMC5	5.46E+06	3.20E+06	11	4.20E+04	6.06E+04	10
AEBP1	6.11E+06	3.14E+06	11	5.46E+05	1.14E+06	10
POTEE	6.34E+06	4.68E+06	11	9.07E+05	8.45E+05	10
EPHX1	7.50E+06	5.46E+06	11	1.85E+04	1.81E+04	10
PGM3	6.63E+06	3.62E+06	11	1.57E+05	1.63E+05	10
ANKFY1	5.85E+06	2.63E+06	11	2.51E+05	4.53E+05	10
ADH4	9.21E+06	8.02E+06	11	1.43E+05	2.33E+05	10
TCEAL5	8.19E+06	1.16E+07	11	9.28E+04	1.50E+05	8
HEL-S-41; RGN	7.31E+06	4.27E+06	11	1.53E+05	1.92E+05	10
PTPRN	6.79E+06	3.95E+06	11	2.16E+05	1.92E+05	10
SYN1	7.35E+06	9.29E+06	11	1.08E+06	7.43E+05	10
RUVBL2	8.06E+06	4.34E+06	11	2.46E+04	2.22E+04	9
BDH2	8.99E+06	6.04E+06	11	2.25E+04	2.76E+04	8
KRT31	6.86E+06	7.75E+06	11	1.94E+04	2.42E+04	10
CAPZA2	7.29E+06	5.44E+06	11	2.64E+04	2.86E+04	10
EPHA7	8.06E+06	8.18E+06	11	1.26E+05	1.42E+05	10
Sept11; SEPT11; SEPTIN11	7.97E+06	4.95E+06	11	3.41E+06	5.90E+06	10
NCDN	5.63E+06	4.83E+06	11	1.22E+04	7.55E+03	9
SEPTIN9	7.73E+06	5.04E+06	11	1.31E+05	2.16E+05	10
GNAI2	5.17E+06	3.70E+06	11	1.08E+06	1.78E+06	10
NNMT	1.55E+07	1.60E+07	11	2.25E+04	3.95E+04	7
PCBP1	7.70E+06	5.10E+06	11	2.85E+05	4.84E+05	9
RANBP1	6.03E+06	4.52E+06	11	5.86E+04	6.09E+04	9
RAB6A	7.74E+06	6.13E+06	11	1.62E+04	2.05E+04	10
NEBL	6.29E+06	5.69E+06	11	2.05E+04	1.39E+04	8
PALS2	8.66E+06	7.93E+06	11	3.81E+04	4.61E+04	10
RAB7A	7.32E+06	6.39E+06	11	6.01E+04	3.80E+04	10
CDH6	4.86E+07	9.12E+07	11	1.17E+05	1.44E+05	10
DARS1	8.55E+06	7.02E+06	11	4.26E+04	3.37E+04	9
KPNB1	7.00E+06	3.94E+06	11	1.29E+05	2.66E+05	10
HNMT	6.75E+06	3.39E+06	11	4.83E+04	4.47E+04	10
HSPB1	6.47E+06	3.63E+06	11	1.00E+05	1.28E+05	10
ACADM	7.52E+06	9.86E+06	11	1.53E+05	3.62E+05	9
MAP4	5.59E+06	3.85E+06	11	5.84E+04	6.51E+04	10
ANXA11	5.72E+06	3.29E+06	11	7.71E+05	1.05E+06	10
TPD52	6.14E+06	4.21E+06	11	2.15E+04	2.40E+04	7
RAP1GDS1	6.09E+06	4.02E+06	11	9.99E+03	7.37E+03	9
NQO2	8.56E+06	6.65E+06	11	1.11E+06	1.08E+06	10
COPB2	7.86E+06	7.66E+06	11	7.95E+05	1.42E+06	10
ADGRL1	7.89E+06	7.20E+06	11	3.22E+04	4.25E+04	10
ACTN1	5.45E+06	1.70E+06	11	2.18E+05	4.25E+05	10
VASN	5.65E+06	2.87E+06	11	4.99E+05	7.25E+05	10
OPCML	6.90E+06	4.88E+06	11	6.41E+04	6.36E+04	10
MB	4.02E+07	9.47E+07	11	1.32E+06	1.79E+06	10
LMNA	7.71E+06	5.24E+06	11	3.80E+04	2.67E+04	6
GDA	6.06E+06	3.35E+06	11	2.74E+04	2.06E+04	9
CPB2	5.20E+06	3.53E+06	11	3.07E+04	2.43E+04	10
CGREF1 (Iso 5)	7.19E+06	6.32E+06	11	8.12E+04	7.46E+04	8
MEGF10	5.46E+06	3.64E+06	11	2.25E+04	2.97E+04	9
PSMD1	6.54E+06	4.43E+06	11	4.46E+04	9.35E+04	10
COLEC12	5.21E+06	2.36E+06	11	1.29E+06	2.24E+06	10
YARS1	6.02E+06	3.90E+06	11	8.31E+04	9.00E+04	10
GMFB	6.05E+06	3.17E+06	11	2.23E+05	2.98E+05	9
HNRNPA2B1	8.17E+06	6.29E+06	11	1.03E+05	6.02E+04	10
TUBB4B	1.28E+07	1.38E+07	11	5.83E+05	1.67E+06	10
GSTM5	4.72E+06	2.71E+06	11	1.62E+04	2.25E+04	6
CDH15	5.28E+06	2.97E+06	11	2.90E+04	2.52E+04	8
DES	1.02E+07	9.41E+06	11	9.01E+04	1.27E+05	10
PABPC1	5.68E+06	3.30E+06	11	1.84E+05	2.59E+05	10
IGHV5-51	1.28E+07	1.60E+07	11	1.53E+05	1.97E+05	10
FLNB	3.84E+06	3.31E+06	11	2.62E+04	1.65E+04	5
DDB1	1.06E+07	1.82E+07	11	2.47E+05	4.65E+05	10
ITIH4	4.93E+06	4.94E+06	10	8.97E+03	1.16E+04	6
ETFA	1.05E+07	1.18E+07	11	2.09E+04	1.42E+04	9
IGKV3D-15	1.17E+07	1.67E+07	11	2.67E+06	4.22E+06	9
USO1	5.20E+06	4.10E+06	11	5.48E+04	6.23E+04	9
XPO7	7.26E+06	8.93E+06	11	6.14E+05	8.88E+05	10
RNASET2	4.05E+06	2.38E+06	11	8.59E+04	9.13E+04	10
VAT1	6.24E+06	5.04E+06	11	2.54E+04	2.47E+04	9
ECM2	7.62E+06	6.06E+06	11	2.46E+04	2.14E+04	7
FTL	8.21E+06	5.81E+06	11	1.31E+04	6.09E+03	7
CRABP2	9.16E+06	1.26E+07	11	2.83E+04	5.04E+04	10
SNX2	5.49E+06	3.36E+06	11	2.55E+04	2.81E+04	10
PLEC	1.58E+07	1.89E+07	11	2.60E+05	2.97E+05	6
APOM	7.62E+06	7.16E+06	11	2.77E+04	2.78E+04	10
C11orf54	8.67E+06	8.53E+06	11	1.09E+05	1.85E+05	10
Eif5a; EIF5A; LOC101358224; LOC111161197; LOC117799153; LOC496181	6.57E+06	4.14E+06	11	2.17E+05	2.74E+05	10
HNRNPC	4.45E+06	2.35E+06	11	7.73E+04	7.05E+04	10
CPQ	4.04E+06	2.26E+06	11	1.51E+05	2.33E+05	10
NFASC	4.86E+07	1.44E+08	11	2.20E+05	4.62E+05	9
CKM	1.10E+07	1.98E+07	11	1.40E+05	1.82E+05	10
CUTA	4.97E+06	4.37E+06	11	6.80E+04	8.21E+04	9
GPX1	5.60E+06	3.88E+06	11	3.76E+04	4.88E+04	8
CRYAB	5.12E+06	2.40E+06	11	5.40E+04	5.36E+04	10
CCT5	6.51E+06	6.14E+06	11	4.01E+05	3.70E+05	10
EHD2	4.20E+06	1.72E+06	11	2.35E+04	1.91E+04	10
RTN4R	2.14E+07	5.79E+07	11	3.18E+04	2.15E+04	10
BASP1	5.84E+06	3.58E+06	9	9.02E+04	6.21E+04	4
PAICS	6.22E+06	4.52E+06	11	1.79E+04	2.63E+04	10
F13B	6.36E+06	5.43E+06	11	3.30E+04	3.15E+04	10
MMP9	6.44E+06	3.70E+06	11	4.11E+05	6.37E+05	10
LGALS3	7.47E+06	5.41E+06	11	1.92E+04	1.89E+04	9
SLC1A3	8.11E+06	9.19E+06	11	2.98E+04	3.81E+04	10
HNRNPH1	5.10E+06	3.58E+06	11	1.38E+05	1.53E+05	10
MRC2	4.86E+06	3.22E+06	11	3.28E+04	1.60E+04	9
AIMP1	7.41E+06	4.88E+06	11	8.70E+04	1.94E+05	10
XPNPEP1	5.56E+06	4.54E+06	11	3.50E+04	4.82E+04	10
PEPD	5.40E+06	2.94E+06	11	3.88E+04	2.52E+04	10
GRHPR	9.68E+06	1.10E+07	11	1.60E+06	1.95E+06	10
MARS1	5.99E+06	6.04E+06	11	1.87E+04	2.60E+04	9
ACAT1	4.76E+06	5.71E+06	11	3.47E+05	3.28E+05	7
FSTL1	4.24E+06	2.46E+06	11	1.17E+05	8.55E+04	10
SKP1	5.70E+06	4.84E+06	11	3.23E+04	2.20E+04	9
AP1B1	1.80E+07	2.62E+07	11	3.21E+04	3.35E+04	10
PRPH	5.78E+06	3.22E+06	11	2.18E+05	5.00E+05	10
HYOU1	4.15E+06	2.49E+06	11	1.58E+05	2.39E+05	10
CYB5R2	4.35E+06	1.59E+06	11	6.79E+04	1.22E+05	10
ADIRF	4.61E+06	2.95E+06	11	1.46E+04	1.35E+04	6
SEZ6L2	9.48E+06	9.68E+06	11	9.40E+04	1.70E+05	10
MMP2	6.09E+06	4.30E+06	11	4.90E+04	4.05E+04	10
SLC4A1	7.06E+06	5.82E+06	11	6.20E+04	5.52E+04	10
MASP1	9.43E+06	1.31E+07	11	7.87E+04	1.12E+05	10
SOD2	4.29E+07	8.22E+07	11	2.97E+04	2.89E+04	10
IDH2	1.12E+07	1.16E+07	11	1.20E+05	9.58E+04	10
BPNT1	6.49E+06	4.46E+06	11	6.76E+04	9.44E+04	9
KRT13	4.49E+06	4.25E+06	11	6.83E+04	8.84E+04	9
APEH	5.74E+06	4.18E+06	11	5.17E+04	6.18E+04	9
CNP	3.84E+06	2.32E+06	11	4.20E+04	3.92E+04	10
PTPA	5.70E+06	3.11E+06	11	5.20E+04	5.25E+04	10
ACTR2	5.29E+06	2.77E+06	11	8.21E+04	1.58E+05	9
PTPRS	9.06E+06	1.82E+07	11			0
GYG1	6.58E+06	6.12E+06	11	7.98E+03	7.85E+03	6
ACAT1	1.03E+07	1.39E+07	11	2.67E+04	3.53E+04	10
DPP7	4.09E+06	2.09E+06	11	4.72E+04	3.88E+04	10
CRMP1	4.71E+06	3.66E+06	11	5.70E+04	7.96E+04	8
PALM	5.78E+06	3.90E+06	11	2.01E+05	2.70E+05	8
CSE1L	4.79E+06	3.61E+06	11	8.95E+04	6.79E+04	10
GNAO1	5.62E+06	4.26E+06	11	2.30E+04	1.97E+04	8
IGKV4-1	7.94E+06	9.79E+06	11	4.90E+04	5.72E+04	10
CADM3	4.00E+06	3.25E+06	11	1.00E+05	1.35E+05	10
JUP	5.29E+06	3.32E+06	11	2.65E+05	3.75E+05	9
SHMT1	3.46E+06	1.44E+06	11	9.43E+04	1.57E+05	10
PLIN1	5.58E+06	4.45E+06	11	1.50E+05	1.41E+05	8
RBP1	9.63E+06	1.19E+07	11	1.37E+05	1.78E+05	10
SSB	4.71E+06	2.03E+06	11	9.21E+04	1.69E+05	9
CDC42EP4	6.82E+06	5.26E+06	11	3.86E+04	4.05E+04	7
OPLAH	5.40E+06	4.14E+06	11	8.05E+04	1.45E+05	10
HSP90AB2P	5.17E+06	3.77E+06	11	2.74E+04	1.42E+04	8
PPP5C	6.28E+06	5.07E+06	11	4.68E+04	4.70E+04	10
CYCS	5.78E+06	4.70E+06	11	6.43E+04	1.16E+05	9
GNPDA1	4.37E+06	3.25E+06	11	3.10E+04	3.25E+04	8
RABGGTA	3.49E+06	2.16E+06	11	2.61E+04	1.31E+04	8
QPRT	3.67E+07	1.04E+08	11	1.45E+05	2.40E+05	10
ISLR	4.64E+06	3.27E+06	11	7.31E+04	1.27E+05	10
TP53I3	4.79E+06	2.32E+06	11	9.23E+05	1.14E+06	10
KRT13	8.02E+07	1.34E+08	3	8.27E+03		1
CBS	1.10E+07	9.89E+06	11	2.53E+04	2.05E+04	10
GCLC	5.04E+06	3.36E+06	11	2.33E+05	6.34E+05	9
SYNE1	3.49E+06	2.84E+06	11	4.90E+04	7.58E+04	6
PNPO	4.81E+06	4.16E+06	11	2.02E+07	2.33E+07	10
GPLD1	4.29E+06	3.61E+06	11	7.89E+04	1.72E+05	10
SERPINA5	6.11E+06	5.10E+06	11	1.23E+06	2.04E+06	10
FBP1	1.10E+07	1.18E+07	11	6.65E+04	6.57E+04	10
ALDH1A2	6.11E+06	5.27E+06	11	4.84E+04	6.36E+04	9
TKFC	5.04E+06	4.16E+06	11	1.12E+05	1.95E+05	10
MFGE8	3.53E+06	2.79E+06	11	7.65E+04	1.82E+05	9
KRT75	6.45E+06	8.13E+06	11	3.26E+05	5.62E+05	10
EIF4A1	5.97E+06	4.23E+06	11	7.06E+05	1.15E+06	10
LRRC47	8.56E+06	9.38E+06	11	6.04E+04	8.94E+04	10
GPX3	6.28E+06	4.46E+06	11	3.29E+04	3.32E+04	8
FETUB	5.30E+06	5.60E+06	11	2.78E+04	2.44E+04	9
DCXR	4.35E+06	3.75E+06	11	6.37E+04	1.10E+05	5
CFL2; CFL2b; LOC101358143; LOC111160529	5.01E+06	2.87E+06	11	8.29E+04	7.82E+04	10
HAAO	1.23E+07	1.54E+07	11	2.90E+04	3.56E+04	10
BPNT2	4.51E+06	3.35E+06	11	3.16E+04	2.93E+04	9
CDC37	4.96E+06	4.03E+06	11	5.71E+04	5.84E+04	9
ME1	4.67E+06	2.55E+06	11	2.02E+04	2.04E+04	10
PRKCSH	4.33E+06	2.90E+06	11	1.75E+04	1.88E+04	10
SARS1	1.02E+07	1.22E+07	11	3.18E+04	3.93E+04	9
GFRA2	5.25E+06	3.59E+06	11	2.07E+04	1.29E+04	9
ENPP6	5.14E+06	4.19E+06	11	4.67E+04	6.41E+04	10
SNCA	5.01E+06	4.07E+06	11	5.80E+04	7.21E+04	9
TUBA1C	8.46E+06	1.05E+07	11	6.29E+04	8.42E+04	8
HSPA1L	5.40E+06	4.15E+06	11	1.51E+05	2.17E+05	9
ENDOD1	3.48E+06	2.65E+06	11	8.25E+04	1.26E+05	10
ARG1	7.74E+06	9.47E+06	11	8.61E+04	8.93E+04	9
GART	6.14E+06	5.24E+06	11	2.11E+04	2.44E+04	9
CYRIB	4.61E+06	3.48E+06	11	3.74E+04	6.52E+04	9
PRCP	4.13E+06	2.84E+06	11	8.63E+04	8.50E+04	8
BSG	4.35E+06	3.49E+06	11	2.75E+04	2.02E+04	9
CS	3.41E+06	2.46E+06	11	1.65E+04	1.76E+04	7
GAP43	4.50E+06	3.94E+06	11	1.78E+05	2.32E+05	10
TPM3	5.22E+06	4.43E+06	11	3.36E+04	2.84E+04	10
ICAM5	5.76E+06	6.34E+06	11	3.12E+04	3.15E+04	10
THBS2	3.66E+06	1.62E+06	11	1.34E+04	1.14E+04	10
ACOT2	5.02E+06	5.09E+06	11	1.09E+06	1.50E+06	10
HMGCS2	5.14E+06	5.63E+06	11	1.53E+04	1.27E+04	9
TXNL1	3.61E+06	2.48E+06	11	1.87E+05	2.81E+05	10
RACK1	7.03E+07	2.22E+08	11	2.31E+07	2.09E+07	10
HNRNPA3; HNRPA3	3.92E+06	2.80E+06	11	2.76E+04	4.31E+04	10
DDX39B	5.41E+06	4.31E+06	11	3.69E+05	6.34E+05	10
AOX1	6.40E+06	8.27E+06	11	1.53E+05	1.71E+05	10
GANAB	5.28E+06	3.14E+06	11	7.94E+04	8.99E+04	9
SLC1A2	4.33E+06	3.21E+06	11	1.33E+05	1.66E+05	8
FBLN1	5.36E+06	6.33E+06	11	4.11E+04	7.46E+04	9
OMD	4.03E+06	2.96E+06	11	2.34E+04	3.37E+04	9
HEL-S-298; PTGR2	4.09E+06	2.60E+06	11	6.68E+04	6.80E+04	10
IGFALS	6.41E+06	5.43E+06	11	1.47E+04	1.54E+04	9
DLD	4.17E+06	2.46E+06	11	2.41E+04	2.43E+04	9
CES1	4.37E+07	1.22E+08	11	9.87E+06	1.86E+07	10
ADAMTS1	4.52E+06	3.65E+06	11	5.31E+06	1.45E+07	8
GOLM1	4.59E+06	5.04E+06	11	1.91E+05	3.13E+05	10
BAG3	4.64E+06	3.56E+06	11	3.34E+04	1.90E+04	9
FKBP3	8.66E+06	1.23E+07	11	3.14E+04	4.04E+04	10
SEMA4D	5.00E+06	6.29E+06	11	2.18E+04	1.25E+04	10
CPPED1	4.12E+06	3.31E+06	11	6.47E+04	6.64E+04	10
SCRN2	4.14E+06	1.93E+06	11	9.18E+03	9.33E+03	9
ATP4A	3.67E+06	2.78E+06	11	4.26E+05	6.17E+05	9
COMT	7.36E+06	1.14E+07	11	1.67E+04	1.61E+04	8
FBLN2	4.84E+06	4.40E+06	11	8.65E+03	5.25E+03	9
POMGNT1	3.45E+06	1.71E+06	11	2.87E+04	1.92E+04	10
UBA6; UBE1L2	4.69E+06	3.26E+06	11	7.75E+04	1.17E+05	9
CPVL	4.91E+06	4.51E+06	11	1.09E+04	8.11E+03	8
VSIG4	3.84E+06	3.29E+06	11	9.68E+04	1.12E+05	10
CNTFR	4.99E+06	5.75E+06	11	1.10E+05	2.04E+05	9
IGFBP5	4.33E+06	4.47E+06	11	2.33E+04	3.21E+04	10
AP2A2	2.88E+06	1.74E+06	11	2.47E+04	4.03E+04	8
HSPA2	3.69E+06	3.57E+06	11	3.11E+04	4.54E+04	10
TUBB3	8.77E+06	1.00E+07	11	1.49E+05	2.99E+05	9
F9	3.97E+06	3.43E+06	11	4.95E+04	6.68E+04	9
ASAH1	5.18E+06	5.34E+06	11	1.84E+04	1.79E+04	9
FOLR1	6.17E+06	8.77E+06	11	2.07E+05	2.21E+05	10
SEZ6	3.21E+06	1.89E+06	11	3.24E+05	8.16E+05	9
PSMB1	3.72E+06	2.43E+06	11	5.04E+04	9.73E+04	10
TPM2	3.49E+06	2.32E+06	11	1.62E+04	2.25E+04	10
SPP1	2.89E+06	1.47E+06	10	1.18E+05	1.51E+05	6
AKR1C2	4.20E+06	3.41E+06	11	3.57E+04	5.01E+04	8
AKR7A3	4.33E+06	3.47E+06	11	4.42E+04	7.63E+04	10
QARS1	4.57E+06	3.33E+06	11	7.20E+04	9.32E+04	10
PSMB4	5.33E+06	4.14E+06	11	7.15E+04	1.36E+05	7
TMOD1	5.53E+06	4.97E+06	11	2.21E+04	2.01E+04	10
CTSH	3.33E+06	1.59E+06	11	6.67E+04	6.93E+04	8
ECHDC3	3.78E+06	2.51E+06	11	1.73E+04	1.51E+04	10
GMPR	3.16E+06	2.53E+06	11	3.11E+04	2.66E+04	10
SPR	3.47E+06	2.50E+06	11	7.01E+04	1.08E+05	9
NUCKS1	2.95E+06	1.88E+06	11	1.75E+05	3.50E+05	9
CACHD1	5.39E+06	5.93E+06	11	8.16E+05	1.75E+06	10
AIFM1	3.38E+06	2.67E+06	11	2.99E+04	3.15E+04	9
ECHDC1	3.60E+06	2.66E+06	11	1.19E+04	1.67E+04	9
ATP6V1B2	3.86E+06	2.59E+06	11	1.61E+04	1.50E+04	9
PITHD1	2.38E+06	1.56E+06	11	8.44E+04	1.28E+05	9
TPT1	2.77E+06	2.26E+06	11	1.65E+05	2.93E+05	9
CADM2	3.48E+06	2.34E+06	11	6.33E+04	7.98E+04	9
BPGM	3.42E+06	2.39E+06	11	3.23E+04	3.04E+04	10
PSMA1	4.36E+06	3.34E+06	11	2.80E+04	2.09E+04	8
ANXA2P2	1.13E+07	1.99E+07	11	2.04E+04	1.38E+04	6
PHPT1	3.85E+06	2.87E+06	11	2.75E+05	3.29E+05	10
TPP1	4.09E+06	3.75E+06	11	1.72E+04	1.23E+04	9
GSTT1	4.25E+06	3.87E+06	11	3.05E+04	2.70E+04	10
EML2	5.20E+06	3.64E+06	11	1.10E+05	1.53E+05	10
KRT17	3.42E+06	3.00E+06	11	7.83E+06	8.99E+06	10
APEX1	3.52E+06	2.36E+06	11	1.06E+05	1.37E+05	10
CLTCL1	3.45E+06	2.51E+06	8	5.80E+05	4.32E+05	4
PDLIM5	3.23E+06	2.35E+06	11	6.56E+04	8.86E+04	10
EIF3B	3.59E+06	1.88E+06	11	4.67E+04	4.04E+04	9
PRKAR2A	3.69E+06	2.68E+06	11	1.30E+04	1.30E+04	10
DSC2	2.53E+06	2.23E+06	11	5.28E+04	7.46E+04	8
PTBP1	5.57E+06	7.73E+06	11	2.99E+04	3.42E+04	9
PCK1	3.22E+06	2.53E+06	11	1.24E+05	1.53E+05	10
PPP1CB	3.29E+06	2.97E+06	11	3.19E+04	3.00E+04	10
ALDOB	2.02E+07	5.19E+07	11	1.52E+06	1.46E+06	10
KRT8	4.10E+06	4.41E+06	11	1.90E+05	2.24E+05	4
MRC1	3.69E+06	2.24E+06	10	6.61E+03	3.23E+03	6
CNN3	3.21E+06	2.69E+06	11	4.43E+04	5.66E+04	8
CRIP2	3.58E+06	3.51E+06	11	1.99E+04	1.67E+04	9
C4BPB	3.88E+06	3.76E+06	11	1.34E+04	8.57E+03	7
FBXO2	5.04E+06	5.65E+06	11	3.83E+04	3.63E+04	10
CRKL	2.80E+06	1.96E+06	11	1.02E+05	1.67E+05	10
TPM3	3.18E+06	2.99E+06	11	7.00E+04	1.45E+05	10
HARS1	3.39E+06	2.31E+06	11	3.08E+04	2.89E+04	10
TPM1	3.08E+06	2.06E+06	11	7.51E+03	2.80E+03	5
SNCB	3.10E+06	2.40E+06	11	4.16E+04	4.09E+04	6
PPP3CA	3.72E+06	3.22E+06	11	4.90E+04	5.08E+04	10
FCN3	3.79E+06	3.26E+06	11	2.28E+05	4.73E+05	10
PCCA	5.32E+06	5.92E+06	11	1.50E+06	4.01E+06	10
PPP2CA	3.54E+06	3.29E+06	11	2.84E+05		1
ACAT2	3.41E+06	2.57E+06	11	7.01E+04	1.03E+05	10
NID2	2.76E+06	2.19E+06	11	2.21E+04	2.17E+04	8
BIN1	3.07E+06	2.44E+06	11	2.54E+04	4.36E+04	10
HADH	4.24E+06	5.22E+06	11	6.10E+04	1.02E+05	10
CDV3	2.87E+06	1.80E+06	11	1.46E+05	1.48E+05	10
LOC114109396; RPS3	2.21E+07	6.25E+07	11	2.90E+04	2.43E+04	9
ASRGL1	3.29E+06	2.68E+06	11	2.06E+05	3.32E+05	8
NRXN1	2.87E+06	1.72E+06	11	5.18E+04	3.40E+04	5
ADH1A	7.73E+06	7.17E+06	10	9.19E+04	1.60E+05	5
SLC3A2	2.94E+06	2.11E+06	11	5.69E+04	8.26E+04	10
HGFAC	2.45E+06	1.18E+06	11	2.77E+04	3.49E+04	10
PRMT5	3.67E+06	2.65E+06	11	7.39E+04	6.03E+04	9
PPP2CB	3.33E+06	3.00E+06	11	2.01E+05	2.53E+05	10
PLS1	4.10E+06	3.82E+06	11	4.28E+04	5.19E+04	10
MTAP	3.05E+06	2.31E+06	11	8.21E+04	9.25E+04	10
ABAT	5.76E+06	8.24E+06	11	3.93E+04	4.83E+04	9
CAPNS1	2.94E+06	1.92E+06	11	2.32E+04	3.03E+04	9
FN3K	2.95E+06	2.23E+06	11	6.16E+04	1.08E+05	9
ATG7	2.18E+06	1.35E+06	11	5.56E+04	7.42E+04	10
GALM	3.11E+06	2.68E+06	11	4.77E+04	8.33E+04	10
XRCC6	3.43E+06	2.27E+06	11	6.92E+04	8.33E+04	10
SLITRK1	6.69E+06	1.00E+07	11	1.21E+04	8.08E+03	9
MPI	2.85E+06	1.88E+06	11	1.33E+05	2.39E+05	10
DCTN2	4.52E+06	5.96E+06	11	1.61E+04	2.20E+04	8
COMP	2.75E+06	2.31E+06	11	6.86E+04	1.22E+05	9
EIF6	9.08E+06	1.34E+07	11	2.86E+05	6.35E+05	6
FDPS	3.03E+06	2.45E+06	11	1.41E+04	9.23E+03	10
TUBA8	4.24E+06	6.82E+06	11	2.61E+04	2.21E+04	9
PDLIM3	3.37E+06	3.31E+06	11	2.71E+05	4.28E+05	9
TPM4	1.14E+07	2.76E+07	11	3.19E+04	4.04E+04	4
HMGB1	2.33E+06	2.32E+06	11	4.21E+04	4.53E+04	7
NADK2	2.81E+06	1.80E+06	11	1.56E+04	1.37E+04	9
ARHGDIB	2.63E+06	1.63E+06	11	4.30E+04	4.74E+04	9
AGA	2.12E+06	1.04E+06	11	2.53E+04	1.57E+04	10
NUDT5	3.72E+06	3.06E+06	11	4.31E+04	6.46E+04	7
FSTL4	2.63E+06	1.76E+06	11	4.07E+04	4.01E+04	10
DYNC1I2	2.31E+06	1.33E+06	11	1.64E+04	1.29E+04	8
RGMB	2.23E+06	1.79E+06	11	6.28E+04	8.06E+04	9
MAT2A	2.58E+06	2.21E+06	11	2.34E+04	2.68E+04	8
ECHS1	5.72E+06	1.10E+07	11	5.20E+04	4.09E+04	10
PI16	2.92E+07	9.05E+07	11	1.52E+04	1.27E+04	9
EPB41L3	7.37E+06	7.71E+06	11	1.36E+06	3.54E+06	9
CACNA2D1	1.44E+06	1.10E+06	9	7.54E+04	3.04E+04	2
MYL6	7.95E+06	1.10E+07	11	1.32E+05	2.35E+05	9
TUBB4A	3.57E+06	2.57E+06	11	1.88E+04	1.68E+04	9
FMOD	2.24E+06	2.03E+06	11	3.77E+04	2.76E+04	10
ATP1A3	3.61E+06	3.33E+06	11	1.19E+05	1.64E+05	9
CAPZA1	4.36E+06	4.52E+06	11	2.25E+04	1.56E+04	8
GRN	2.73E+06	2.81E+06	11	3.19E+04	1.95E+04	4
GRN	2.23E+06	2.71E+06	11	3.66E+04	4.64E+04	10
NME1	2.78E+06	2.32E+06	11	1.64E+04	2.43E+03	2
CANX	6.10E+06	1.24E+07	11	6.71E+04	6.94E+04	10
SPOCK1	3.00E+06	2.45E+06	11	1.10E+05	1.39E+05	8
GPC1	2.03E+06	1.17E+06	11	2.66E+06	7.45E+06	9
CRYM	2.58E+06	2.23E+06	11	4.82E+04	1.12E+05	9
IGLC6	1.85E+06	5.65E+05	9	6.33E+04	8.37E+04	5
LTA4H	1.63E+06	1.22E+06	11	2.99E+04	3.00E+04	8
RAB14	3.00E+06	2.12E+06	11	1.55E+05	3.40E+05	8
NAGK	2.51E+06	1.86E+06	11	1.86E+05	2.82E+05	10
APPL1	2.26E+06	2.14E+06	11	1.61E+06	4.34E+06	8
MAT2B	3.64E+06	4.90E+06	11	4.87E+04	3.78E+04	3
PITPNA	2.51E+06	2.07E+06	11	2.07E+04	2.38E+04	8
PTPRD	1.85E+06	1.16E+06	11	1.47E+04	2.13E+04	6
BCAM	6.21E+06	9.74E+06	11	3.06E+04	2.77E+04	10
PGAM2	2.28E+06	1.88E+06	11	9.43E+04	1.84E+05	6
HDDC2	1.96E+06	1.25E+06	11	4.26E+04	5.01E+04	5
CLSTN1	1.65E+06	1.09E+06	11	3.23E+04	6.02E+04	6
IGKV1-8	2.30E+06	3.32E+06	11	3.03E+04	2.71E+04	5
DBI	1.77E+06	7.32E+05	5			0
Eif4a2; EIF4A2; LOC101352730; LOC111152235	3.04E+07	9.32E+07	11	1.37E+04	1.12E+04	7
WARS1	2.79E+06	2.35E+06	11	9.74E+03	5.85E+03	10
NCAM1	2.07E+06	1.29E+06	11	5.07E+03	1.67E+03	3
TTC38	3.26E+06	1.93E+06	11	1.02E+06	1.58E+06	10
LPP	1.63E+06	9.92E+05	11	2.96E+04	2.57E+04	9
QDPR	1.31E+06	1.10E+06	5	2.07E+03		1
SEPTIN6	2.36E+06	1.68E+06	11	4.07E+04	6.25E+04	10
EIF3I	1.78E+06	8.47E+05	11	4.31E+05	8.63E+05	10
CD99L2	1.46E+06	1.29E+06	11	4.82E+04	9.17E+04	10
POTEJ	2.10E+06	1.77E+06	11	1.21E+04	1.29E+04	6
TPM4	2.60E+06	2.16E+06	11	1.00E+04	8.76E+03	7
UBE2V2	2.09E+06	1.68E+06	11	9.49E+04	1.85E+05	6
GAA	1.97E+06	1.19E+06	11	1.46E+04	1.55E+04	10
IGKV1-33	3.96E+06	5.78E+06	11	3.18E+04	2.76E+04	9
PPT1	2.13E+06	2.07E+06	11	1.80E+04	1.70E+04	8
WBP2	2.02E+06	1.57E+06	11	2.81E+04	4.41E+04	8
FHL1	2.14E+06	2.49E+06	11	5.06E+05	5.74E+05	7
ZYX	3.68E+06	7.26E+06	11	5.25E+04	4.20E+04	10
LRRC4B	1.65E+06	1.26E+06	11	3.92E+04	3.39E+04	10
NIT1	2.68E+06	2.28E+06	11	2.15E+04	3.04E+04	10
DDT	4.13E+06	5.12E+06	11	1.31E+05	1.08E+05	2
ACTR1A	2.96E+06	3.07E+06	11	1.93E+04	1.80E+04	9
GATM	2.80E+06	3.33E+06	11	3.97E+04	6.83E+04	10
SNX6	2.18E+06	1.63E+06	11	1.95E+04	1.09E+04	7
GPT	2.25E+06	2.34E+06	11	3.52E+07	6.52E+07	10
NEB	1.60E+06	7.66E+05	11	1.89E+04	2.03E+04	3
GSTM1	5.16E+06	6.25E+06	11	3.75E+04	8.48E+04	7
UCHL3	2.03E+06	1.35E+06	11	4.19E+04	5.37E+04	10
SORBS1	2.34E+06	2.09E+06	11	3.38E+04	3.16E+04	6
ACTN3	2.82E+06	3.21E+06	11	1.72E+04	2.09E+04	8
IGHV3-66	1.93E+06	1.57E+06	11	2.73E+04	2.81E+04	6
PSMB5	2.61E+06	2.30E+06	11	2.06E+04	2.09E+04	9
ETFB	2.22E+06	3.02E+06	11	4.14E+04	6.26E+04	6
DDX39A	2.01E+06	1.06E+06	11	8.28E+04	1.20E+05	7
ACTR1B	1.67E+06	1.45E+06	11	6.79E+04	1.10E+05	10
IGHV3-53	1.03E+06	7.52E+05	3			0
PHYHD1	1.42E+06	1.02E+06	11	2.75E+04	4.30E+04	6
CANT1	1.37E+06	9.44E+05	11	2.28E+04	2.66E+04	9
AEBP1	1.41E+06	9.22E+05	7	2.69E+04	7.45E+03	2
POTEKP	2.32E+06	2.88E+06	11	5.53E+04	6.22E+04	6
ANK1	2.33E+06	2.68E+06	11	6.73E+03	6.87E+03	3
PBLD	1.94E+06	1.93E+06	11	2.57E+04	1.99E+04	5
BTD	1.58E+06	1.16E+06	7			0
PCBP2	3.01E+06	1.77E+06	11	1.19E+04	1.18E+04	6
GNPDA2	1.78E+06	1.10E+06	11	2.13E+05	4.00E+05	10
MT3	1.81E+06	1.18E+06	11	1.25E+06	1.39E+06	10
IGKV1-17	2.30E+06	2.47E+06	11	1.67E+05	2.61E+05	8
ALDH1B1	2.15E+06	2.16E+06	11	3.22E+04	3.25E+04	10
RHOA	1.70E+06	1.04E+06	11	1.79E+04	9.07E+03	5
ACAA2	7.68E+06	2.02E+07	11	1.12E+05	2.20E+05	10
ENO3	1.28E+06	3.44E+05	4			0
SELENBP1	1.30E+06	9.57E+05	10	2.18E+04	1.44E+04	3
DPYS	1.82E+06	1.67E+06	11	1.36E+05	1.64E+05	9
GLRX	1.58E+06	1.52E+06	11	4.81E+04	6.85E+04	8
GSTT2	2.40E+06	2.47E+06	11	2.58E+04	2.75E+04	10
NT5DC1	1.09E+06	7.35E+05	11	2.83E+04	3.04E+04	9
CYB5R3	1.55E+06	1.34E+06	11	1.99E+04	2.29E+04	7
TGM2	3.15E+06	4.64E+06	11	2.71E+04	2.64E+04	10
NELL2	2.02E+06	2.19E+06	11	1.93E+04	1.45E+04	2
STRAP	1.62E+06	1.46E+06	11	2.69E+04	1.51E+04	8
PRRT2	1.90E+06	1.79E+06	11	6.35E+04	7.54E+04	8
SEMA6D	1.22E+06	7.13E+05	11	9.56E+04	6.62E+04	5
BGN	2.01E+06	1.88E+06	11	3.05E+04	4.43E+04	8
CPNE3	2.19E+06	2.12E+06	11	2.21E+04	1.93E+04	8
GATD3	1.70E+06	2.67E+06	11	3.55E+04	4.14E+04	7
AKR1B10	3.72E+06	6.26E+06	11	2.06E+04	1.20E+04	10
KRT83	4.20E+06	4.97E+06	11	1.76E+05	3.45E+05	7
ENOPH1	1.67E+06	1.50E+06	11	2.50E+05	4.01E+05	9
YWHAE	2.52E+06	2.08E+06	11	3.66E+03	1.53E+03	3
HSPA6	2.17E+06	2.31E+06	11	1.94E+04	1.95E+04	8
ADH1C	9.91E+06	2.71E+07	11	1.81E+04	3.56E+04	10
CCT3	1.04E+06	5.04E+05	9	1.27E+04	9.47E+03	3
PPIA	1.51E+06	1.42E+06	10	3.03E+05	3.35E+05	10
TWF2	1.54E+06	9.96E+05	11	1.58E+04	1.71E+04	9
PKLR	2.21E+06	2.18E+06	11	6.71E+03	4.02E+03	5
MARCKSL1	1.43E+06	1.24E+06	11	1.41E+05	1.57E+05	9
ATP2B2	2.15E+06	1.98E+06	11	9.48E+03	6.71E+03	10
PTK7	2.04E+06	2.81E+06	10	2.84E+05	6.12E+05	5
PSAP	4.05E+06	5.40E+06	6			0
KRT33A	2.24E+06	2.40E+06	11	2.01E+05	4.19E+05	9
NPDC1	3.51E+06	8.17E+06	11	9.45E+03	6.55E+03	5
SHPK	1.31E+06	9.63E+05	11	2.47E+04	3.63E+04	9
MT2A	1.39E+06	9.65E+05	10	8.46E+05	1.05E+06	5
EEF1A2	1.45E+06	1.14E+06	11	1.32E+04	1.23E+04	6
IDS	1.04E+06	7.47E+05	11	9.82E+03	7.98E+03	6
HNRNPU	9.17E+05	5.78E+05	11	3.81E+04	4.08E+04	6
ATP6AP1	1.02E+06	7.25E+05	11	7.27E+03	5.69E+03	6
GMPPB	1.19E+06	9.00E+05	11	6.23E+03	3.18E+03	8
ENPP2	2.04E+06	2.61E+06	11	1.54E+04	6.74E+03	4
AKR1C4	9.96E+05	1.26E+06	11	1.33E+06	2.95E+06	5
PGK2	9.39E+05	4.83E+05	11	2.37E+04	2.31E+04	7
TUBB1	2.27E+06	2.57E+06	10	3.41E+05	9.22E+05	10
NDRG1	2.17E+06	2.92E+06	11	3.60E+04	7.56E+04	7
SPP1	1.14E+06	8.81E+05	11	4.07E+04	2.57E+04	5
SEPTIN7	2.81E+06	5.98E+06	8	4.47E+04	4.82E+04	7
PPP1R7	1.00E+06	1.11E+06	9	7.17E+03	7.24E+03	3
MT1X	1.22E+06	1.03E+06	10	2.29E+05	4.07E+05	10
KCTD12	1.58E+06	2.14E+06	11	8.88E+03	2.83E+03	4
PURA	2.82E+06	4.93E+06	11	2.06E+04	1.84E+04	9
DSP	1.07E+06	5.73E+05	10	6.62E+04	6.88E+04	4
BPNT1	1.00E+06	6.09E+05	11	1.61E+04	1.04E+04	6
TST	5.01E+06	1.01E+07	11	1.55E+04	1.95E+04	6
IGKV1-27	1.17E+06	1.32E+06	11	2.05E+04	1.90E+04	3
SHBG	2.40E+06	5.20E+06	11	1.99E+05	5.17E+05	9
MAP2	1.33E+06	1.25E+06	9	3.23E+04	3.55E+04	4
ZYX	1.27E+06	1.04E+06	11	1.69E+04	1.53E+04	5
LMNA	1.17E+06	1.53E+06	9	1.26E+04		1
PPP1CC	1.96E+06	2.34E+06	11	1.20E+04	1.64E+04	8
IGKV1D-39	2.52E+06	2.88E+06	8	8.56E+03	5.03E+03	6
STIP1	1.38E+06	2.26E+06	11	7.39E+03	3.44E+03	5
RAB1B	1.48E+06	1.48E+06	11	4.51E+03	1.96E+03	6
HIBADH	1.50E+06	1.75E+06	11	8.45E+03	7.20E+03	8
NRXN3	1.86E+06	2.98E+06	11	1.46E+04	2.36E+04	8
IGHV2-70D	2.25E+06	3.52E+06	11	6.91E+04	9.68E+04	8
GSTA2	1.37E+06	8.75E+05	11	4.33E+04	7.32E+04	8
GATM	7.91E+05		1			0
SRM	1.16E+06	1.29E+06	11	2.31E+04	3.43E+04	8
GLOD4	9.23E+05		1			0
TUBB8	9.86E+05	6.95E+05	11	2.21E+04	2.40E+04	6
NAXD	2.14E+06	2.77E+06	11	1.00E+04	1.24E+04	9
BHMT2	9.69E+05	1.28E+06	11	8.13E+04	1.30E+05	8
FABP5P3	8.11E+05	7.81E+05	11	9.18E+03	5.26E+03	9
ACLY	9.09E+05	4.31E+05	11	1.50E+05	1.61E+05	6
FAHD2A	6.50E+05	4.67E+05	10	1.63E+04		1
ACTA2	1.61E+06	1.35E+06	10	5.75E+03	4.65E+03	6
FBLN2	9.42E+05	3.28E+05	6			0
CD163	1.51E+06	1.33E+06	5	5.04E+03	2.52E+03	3
CD99	7.54E+05	5.34E+05	11	6.40E+04	6.25E+04	6
EIF4G1	7.91E+05	5.42E+05	11	9.78E+03	3.92E+03	3
AHCYL2	9.07E+05	9.13E+05	11	7.85E+04	1.08E+05	5
ENO1	6.11E+05	3.81E+05	11	4.21E+03	2.65E+03	5
CRB2	1.31E+06	9.99E+05	11	5.66E+03	4.05E+03	10
ARL3	1.26E+06	1.93E+06	11	2.06E+04	2.62E+04	7
LTBP4	9.67E+05	1.11E+06	11	7.62E+03	4.94E+03	7
G6PD	5.66E+05	3.96E+05	6			0
HSPA7	9.68E+05	7.44E+05	10	7.53E+03	6.03E+02	2
HSP90AA2P	1.47E+06	2.65E+06	11	1.16E+04	9.73E+03	6
PLEC	2.70E+06	5.48E+06	10	1.96E+04	1.80E+04	5
GNPTG	1.07E+06	1.02E+06	10	3.44E+04	4.03E+04	9
SIRPB1	6.91E+05	3.97E+05	5			0
PFKL	5.57E+05	3.60E+05	5	3.41E+03	5.90E+02	3
TPD52L2	5.49E+05	1.84E+05	6			0
MACF1	7.30E+05	6.59E+05	11	7.78E+04	7.89E+04	3
TPM2	5.99E+05	6.09E+05	11			0
TUBB2B	7.02E+05	6.59E+05	11	1.12E+04	6.74E+03	6
MMP2	3.48E+06	5.53E+06	9	2.41E+04		1
POTEI	1.81E+06	2.48E+06	10	2.77E+05	5.59E+05	7
RDX	8.72E+05	6.62E+05	9	3.43E+04		1
HSP90AB3P	1.85E+06	2.25E+06	11	3.08E+06	3.00E+06	10
TMOD1	2.74E+06	5.89E+06	7	8.80E+03		1
SCG3	6.59E+05	4.92E+05	11	5.05E+04	3.94E+04	7
GNAI3	7.60E+05	6.71E+05	11	1.26E+04	6.79E+03	5
PTPN11	9.02E+05	7.63E+05	9	2.16E+03		1
DST	1.95E+06	1.97E+06	6			0
PDXP	1.44E+06	1.10E+06	11	4.71E+05	1.02E+06	9
PHYHIPL	8.98E+05	6.40E+05	11	2.24E+04	1.95E+04	8
KLC1	7.93E+05	6.59E+05	6			0
SPTBN1	1.01E+06	1.10E+06	7	4.72E+04	6.46E+04	8
MAP2K1	1.18E+06	1.13E+06	11	1.79E+04	1.12E+04	6
PSMD10	1.05E+06	1.27E+06	11	1.29E+04	9.43E+03	5
DNM1L	9.94E+05	8.67E+05	11	7.84E+03	4.45E+03	5
PFN2	6.17E+05	4.30E+05	11	2.97E+04	3.16E+04	5
DDX1	8.82E+05	7.82E+05	2			0
CTH	4.54E+05	3.15E+05	3			0
SULT1A1	5.99E+05	4.49E+05	11	4.42E+03	6.17E+02	3
ARF4	6.80E+05	4.19E+05	11	3.32E+03	1.35E+03	6
SPAG9	2.37E+05	1.62E+05	2			0
PLEC	6.31E+05	8.53E+05	8	1.93E+04	2.65E+04	5
CA8	1.42E+06	2.36E+06	11	1.84E+04	2.24E+04	8
TXNRD1	2.56E+06		1			0
ACSM2A	1.18E+06	1.61E+06	11	2.20E+05	4.36E+05	10
GSN	1.27E+07	3.06E+07	8	2.48E+03		1
KRT6B	1.78E+06	2.78E+06	11	2.83E+05	3.87E+05	9
HEL-S-129m; PSME1	9.29E+04		1			0
IGLC7	5.34E+05	6.44E+05	11			0
CRTAC1	1.39E+06	1.96E+06	11	8.43E+04	8.79E+04	2
SERPINA1	9.41E+05	5.99E+05	6			0
CDH11	4.41E+05	2.58E+05	10			0
PFKP	3.91E+05	3.30E+05	3			0
EIF4G1	5.71E+05	5.66E+05	2			0
SRI	4.33E+05	2.40E+05	5	4.62E+04	3.41E+04	7
PSAP	8.04E+05	7.10E+05	7			0
HBG2	8.23E+05	9.12E+05	9	7.01E+03	5.02E+03	2
LMNA	4.99E+05	4.21E+05	11	4.18E+05	8.91E+05	5
TNC	4.60E+05	1.69E+05	11	7.14E+03	2.73E+03	4
KTN1	1.14E+06	1.31E+06	10	2.16E+04	2.41E+04	6
MCAM	5.25E+05	5.01E+05	11	7.65E+03		1
IGHD	8.87E+05	8.22E+05	9	8.79E+03	2.95E+03	2
AQP1	1.42E+06	2.96E+06	11	1.23E+04	1.03E+04	8
HSPH1	6.61E+05	6.97E+05	10	6.14E+03	3.14E+03	2
SEZ6	5.30E+05	3.21E+05	9	1.64E+04	1.03E+04	2
RNASE2	7.03E+05	7.42E+05	10	4.07E+04	6.92E+04	5
PPM1A	1.06E+06	2.01E+06	11	7.87E+03	6.43E+03	2
ATP2B4	4.54E+05	8.69E+04	2			0
HNRNPA1	3.54E+05	2.24E+05	6	5.04E+03	1.61E+03	5
DBN1	6.08E+05	6.60E+05	6	5.01E+03	8.10E+01	2
ANK1	4.59E+05		1			0
PPM1B	3.77E+05	1.47E+05	2			0
ACTN4	5.02E+05	5.33E+05	7			0
PGK1	5.25E+05	5.18E+05	11	7.02E+03	2.27E+03	4
EIF5AL1	5.25E+05	5.81E+05	9	8.58E+03	9.02E+03	8
NRXN1	4.96E+05	3.76E+05	3	5.87E+03		1
PGM1	1.10E+06	1.36E+06	11	2.63E+04	2.79E+04	7
DDT	7.14E+05		1			0
PLEC	3.15E+05	2.09E+05	9	5.97E+04	1.27E+05	5
C11orf54	4.50E+05	5.21E+05	4			0
SLC9A3R1	4.36E+05		1	6.66E+03		1
TTN	5.51E+05	4.96E+05	11	8.51E+03	5.32E+03	7
GSN	4.36E+05	3.78E+05	11	1.06E+04	6.66E+03	5
AHCY	4.63E+05	3.58E+05	10	3.06E+03		1
IGLV3-21	3.11E+05	3.19E+05	6	2.68E+04	8.02E+03	2
PAM	6.28E+05	6.70E+05	5	1.90E+04	2.50E+04	4
FN1	2.85E+05	1.19E+05	2			0
GSTM4	5.61E+05	5.81E+05	11	7.10E+04	1.73E+05	9
CNTN1	3.51E+05	1.49E+05	5	2.31E+04	3.30E+03	2
APP	5.97E+05	5.10E+05	6	8.26E+04	5.87E+04	3
ITIH4	4.07E+05	3.85E+05	11	2.06E+04	6.78E+03	3
SIRPA	4.94E+05	6.50E+05	11	2.31E+04	1.14E+04	5
SEPTIN9	2.69E+05	1.37E+05	9	2.03E+05	2.30E+05	5
PTPRN2	3.40E+05	1.55E+05	8			0
SOD2	1.71E+05	2.91E+05	4	2.54E+03		1
UBA1	3.73E+05		1			0
KRT13	1.28E+06	1.59E+06	10	4.89E+03	5.30E+03	8
FBLN1	6.02E+05	8.28E+05	11	8.63E+03	6.68E+03	3
MT1G	8.16E+05	1.44E+06	10	7.80E+04	9.49E+04	9
TUBA8	6.36E+05	7.84E+05	7	3.92E+04	2.86E+04	2
GSTM4	3.60E+05	2.31E+05	4	1.24E+05	1.30E+05	2
ANK1	2.60E+05	1.46E+05	4			0
SEZ6L2	3.19E+05	2.55E+05	11			0
MT1E	3.79E+05	2.93E+05	10	2.49E+05	3.82E+05	8
MAP4	5.87E+05	7.26E+05	11	1.63E+04	2.41E+04	6
EPHA4	1.10E+06	1.97E+06	9	2.81E+05	3.09E+05	4
OPCML	3.21E+05	2.37E+05	11	4.88E+03	1.92E+03	2
AHCYL1	4.08E+05	2.71E+05	9	4.13E+04	3.60E+04	9
IGHV3-23	4.78E+05	2.68E+05	2			0
TCEAL3	4.50E+05	4.44E+05	10	4.22E+04		1
VWF	1.01E+06	1.53E+06	11	1.83E+04	1.07E+04	5
TUBB8B	2.39E+05	1.49E+05	11	2.61E+04	1.03E+04	4
GNAI2	2.82E+05	2.30E+05	9	1.86E+05	3.64E+05	4
CTTN	3.36E+05	2.28E+05	10	1.71E+04	2.05E+04	3
SEC31A	6.35E+05	6.19E+05	8			0
SEPTIN8	3.22E+05	3.55E+04	5	7.35E+03	6.03E+03	4
KRT33B	4.62E+05	3.78E+05	10	2.09E+05	4.54E+05	7
NEO1	2.65E+05	1.84E+05	6	5.49E+03	1.27E+03	2
VCAN	7.62E+05	9.58E+05	11	1.09E+04	3.24E+02	2
LTBP4 (Iso 3)	2.10E+05	7.31E+04	4			0
HLA-C	2.84E+05	1.79E+05	10	8.47E+03	1.15E+03	3
BSG	5.54E+05	4.50E+05	10	3.01E+03	1.98E+03	4
PDLIM5	3.94E+06	7.00E+06	11	1.60E+04	1.47E+04	5
PCBP2	7.92E+05	1.19E+06	8	1.21E+04	1.41E+04	2
MAP4	3.38E+05	1.82E+05	11	4.96E+04	2.42E+04	5
IGKV1D-12	5.30E+05	7.84E+05	10	3.66E+04	4.04E+04	10
RPS27A	4.08E+05	3.49E+05	11	3.37E+03		1
TJP2	3.48E+05	4.45E+05	9	3.25E+03		1
RGMA	3.49E+05	3.12E+05	11	4.32E+04	6.76E+04	7
CAND1	2.58E+05		1			0
AP2M1	2.45E+05		1			0
NAP1L1	5.05E+05	8.74E+05	11	2.34E+06	4.54E+06	10
ECM1	2.41E+05	2.08E+05	7	1.16E+04		1
CHL1	2.74E+05	2.26E+05	8	1.77E+05	2.35E+05	4
Actr2; ACTR2; LOC111147990	4.18E+05	4.39E+05	10	1.29E+04	8.12E+03	2
EPHX2	2.24E+05	2.48E+05	2			0
PLS3	1.71E+05	1.28E+05	5	3.22E+04	1.70E+04	2
SEC31A	3.45E+05	2.52E+05	8			0
GLO1	3.18E+06	2.86E+06	7	3.28E+03	1.12E+03	4
ARF1	2.23E+05	3.70E+04	2			0
MYOF	4.72E+05	3.36E+05	11	2.55E+03	2.19E+03	5
GDI2	1.99E+05	1.94E+05	10	1.25E+04	1.01E+04	7
CCT6A	1.57E+05	8.70E+04	2	8.50E+04	1.32E+05	4
RAB3A	5.55E+05	5.14E+05	11	9.97E+03	8.37E+03	3
AMPD2	2.28E+05	1.94E+04	2			0
HNRNPK	2.13E+05	1.53E+05	6	1.22E+04		1
BIN1	2.23E+05	1.31E+05	6			0
PAM	2.62E+05	1.28E+05	9	1.85E+05	7.08E+04	2
KRT86	6.98E+05	9.57E+05	10	2.71E+03	3.77E+02	4
IGHV3-48	1.58E+05	1.37E+05	8	1.97E+04	9.65E+03	2
ATP2B3	3.03E+05	4.02E+05	11	1.52E+04	1.90E+04	2
ATP2B2	4.64E+05	5.77E+05	10	1.04E+04	7.63E+03	2
DNM1	2.11E+05	5.83E+04	9	3.16E+04		1
WDR1	2.01E+05		1			0
PGAM4	3.48E+05	3.96E+05	11	1.17E+04	8.83E+03	6
PTPRD	2.12E+05		1			0
BTD	1.79E+05	1.11E+05	6			0
NME2P1	4.25E+05	4.69E+05	10	1.07E+06	2.37E+06	8
SLC3A2	1.41E+05	1.14E+05	6	7.84E+04		1
LZTFL1	3.60E+05	3.30E+05	11	7.40E+04	7.32E+04	7
ANK1	2.60E+05	2.18E+05	8			0
SLC39A12	3.27E+05	4.06E+05	7	5.49E+03	3.13E+03	6
RUVBL1	2.11E+05		1			0
CRYZ	1.81E+05	2.08E+05	4			0
TUBA1B	2.54E+05	2.21E+05	10	1.95E+04	1.04E+04	4
GSR	1.42E+05	6.61E+04	8			0
PTPRS	2.05E+05	2.54E+05	8	1.13E+04	1.32E+04	7
PRPH	4.31E+05	4.30E+05	8	9.95E+03	7.40E+03	4
CCT8	3.46E+05	3.35E+05	5	8.76E+03	5.24E+03	3
GSR	1.29E+05	6.97E+04	8			0
RBP1	5.00E+05	7.19E+05	6	6.03E+03	3.69E+03	4
ANK1	3.49E+05	2.95E+05	11	9.43E+03	5.03E+03	2
IGHV3-21	1.55E+05	1.10E+05	5			0
GLUD1	3.95E+05	3.29E+05	5	5.07E+03		1
MBP	1.60E+05	9.31E+04	8	3.74E+03	1.31E+03	2
YWHAZ	3.30E+05	3.37E+05	7	5.66E+03	2.39E+03	2
ELN	1.59E+05	1.48E+05	11	8.76E+03		1
PZP	2.28E+05	1.81E+05	11			0
NPEPPSL1	1.93E+05	1.56E+05	10			0
KLC1	1.65E+05		1			0
AP1B1	2.36E+05	1.42E+05	11	9.97E+04	1.10E+05	6
SPARCL1	1.69E+05		1	5.38E+03	1.55E+03	2
SORBS1	4.72E+05	5.06E+05	7	4.54E+03	2.88E+03	2
HSPA1A	1.79E+05	4.66E+04	5			0
TTN	3.76E+05	4.22E+05	11	3.89E+03	1.84E+03	5
COL3A1	2.76E+05	1.94E+05	7			0
ST13P4	1.16E+05		1			0
NRXN3	2.01E+05	1.38E+05	10	3.01E+03	1.86E+03	2
LOC111149674; Ppp1ca; PPP1CA	2.03E+05	1.83E+05	11	4.88E+03	1.55E+03	8
RAP1GDS1	6.88E+05	1.52E+06	8	5.77E+03		1
LDB3	2.03E+05	1.25E+05	11	9.10E+03	5.79E+03	6
EPB41L2	2.30E+05	2.33E+05	11	1.19E+04	1.08E+04	8
FH	1.91E+05	1.39E+05	11			0
DLD	1.67E+05	2.05E+05	9	2.53E+04	3.03E+04	6
GSTO1	1.13E+05		1	3.40E+04	3.06E+04	3
GPI	3.89E+05	5.71E+05	7	2.63E+03		1
AARS1	2.61E+05	2.70E+05	6			0
DCN	1.57E+05	2.99E+04	3			0
EFEMP1	1.52E+05	8.09E+04	11	1.21E+04	4.83E+03	3
PPIF	3.04E+05	5.02E+05	9	6.73E+03	4.92E+03	7
ROBO1	1.84E+05		1	2.89E+04	1.86E+04	3
PPP1R1B	1.15E+05	1.06E+05	4	8.24E+03	3.13E+03	4
SELENBP1	2.74E+05	2.56E+05	6	6.80E+03		1
HSPH1	2.06E+05	2.96E+05	10	3.82E+03		1
DSP	2.10E+05	2.38E+05	3	4.77E+03	6.21E+02	3
CALD1	2.02E+05	2.17E+05	11	7.62E+04	1.25E+05	6
AKR1C3	1.33E+05	1.06E+05	5			0
ALB	1.22E+05		1	3.64E+03		1
IMPA1	1.04E+05	3.76E+04	7			0
TPM1	4.24E+05	4.71E+05	6			0
CPS1	1.67E+05	1.54E+05	6			0
DDX17	4.34E+05	5.87E+05	11	2.87E+04	3.25E+04	4
NPTN	2.24E+05	1.61E+05	3			0
PGD	1.12E+06		1	4.12E+03	3.66E+02	3
EIF4G1	1.54E+05	1.16E+05	8	5.96E+03	1.66E+03	2
NCAM2	2.37E+05	2.52E+05	9	5.00E+03	3.08E+03	4
HLA-B	1.81E+05	1.92E+05	8			0
MYH14	1.78E+05	1.44E+05	6	4.94E+04	2.82E+04	2
LTBP4 (Iso 2)	2.15E+05	4.27E+05	9	1.00E+04	9.61E+03	6
POMGNT1	1.52E+05	5.13E+04	5			0
EPB41L1	9.35E+04	2.83E+04	7			0
KARS1	1.35E+05	9.51E+04	11	7.98E+04	1.48E+05	5
MPST	2.77E+05	6.05E+05	9	4.96E+03	3.32E+03	4
IGHA2	1.32E+05	2.73E+04	2			0
ETFB	2.49E+05	3.38E+05	7	1.47E+04	4.64E+03	2
SEPTIN9	9.01E+04	4.40E+04	6	1.06E+04		1
MEGF8 (Iso 2)	1.02E+05		1			0
MDH1	1.18E+05	7.15E+04	8			0
CNTNAP4	1.29E+05	9.48E+04	4	8.24E+04	1.06E+05	6
KLC1	3.29E+05	6.00E+05	10	1.34E+04	1.21E+04	4
ACOT1	3.99E+05	6.12E+05	11	1.46E+04	4.67E+03	2
SLC4A1	1.44E+05	1.34E+05	9	4.38E+04	5.42E+04	8
ACOT7	1.12E+05	7.88E+04	10	6.25E+03	3.67E+03	6
NFASC	1.45E+05	8.51E+04	4			0
BSG	1.39E+05	9.42E+04	9	8.60E+03	2.36E+03	3
PGM2	1.48E+05	1.05E+05	9	1.90E+04	2.14E+04	2
CAST	2.27E+05	2.47E+05	7	5.12E+04	9.51E+04	4
RARS1	2.79E+05	2.94E+05	10	5.75E+03	3.57E+03	6
CALD1	1.64E+05	1.32E+05	8	7.18E+03	1.46E+03	2
NID1	1.03E+05	9.76E+04	5			0
LDHA	1.29E+05	1.09E+05	9	4.35E+03		1
EIF4G1	1.18E+05	2.17E+04	2			0
SEMA7A	1.31E+05	1.27E+05	11	6.11E+03	2.80E+03	3
TUBB3	2.48E+05	4.01E+05	7			0
PFKFB2	1.32E+05	4.02E+04	2	2.21E+04	1.47E+04	2
AKAP12	2.04E+05	2.14E+05	7	2.52E+03	6.95E+02	2
ADGRL3	1.35E+05	1.39E+05	4	1.17E+04	1.39E+04	2
COL6A3	1.22E+05	4.32E+04	7	6.30E+03		1
PTPRN2	1.83E+05	1.55E+05	9	6.09E+03	2.46E+03	5
IGHD	2.50E+06	4.72E+06	4			0
TXNRD1	1.14E+05	7.88E+04	6			0
SPTAN1	2.05E+05	3.84E+05	11	7.92E+04	1.19E+05	3
KLC1	9.66E+04	3.94E+04	8			0
MAN2A2	1.86E+05	2.86E+05	9	7.69E+03	5.09E+03	4
NTM	9.08E+04	7.89E+04	9			0
ANXA4	8.71E+04	8.30E+04	3	2.81E+04		1
ITIH1	4.90E+04	2.57E+04	4	1.31E+05	2.06E+05	4
ALDH1A2	1.01E+05	6.91E+04	9	2.55E+04		1
ATP2B1	9.35E+04		1			0
APLP2	2.74E+05	4.68E+05	10	2.33E+03	5.69E+02	3
FCN3	1.51E+05	1.05E+05	6	6.51E+03	9.40E+02	5
NME2	1.47E+05	1.35E+05	7	1.14E+04	8.32E+03	7
PCYT2	1.04E+05	9.42E+04	10	3.11E+03		1
AP2B1	8.51E+04	6.23E+04	5	1.09E+04		1
ADD3	1.50E+05	1.07E+05	7	6.42E+03	6.31E+03	3
YWHAB	8.91E+04		1	9.53E+03		1
ECM2	1.40E+05		1	1.87E+05	2.74E+05	3
SORBS1	2.32E+05	2.21E+05	9	3.39E+03	1.41E+02	3
CAMK2A	1.30E+05	1.79E+05	5	8.58E+03	1.81E+02	2
ALDH7A1	7.33E+04	2.70E+04	3	1.27E+05	2.06E+05	6
PNPO	1.25E+05	1.37E+05	7	3.40E+03		1
PDLIM3	1.16E+05	1.56E+05	9			0
EEF1D	7.43E+04	2.59E+04	4	5.53E+03	2.12E+03	3
UGP2	5.99E+05	1.51E+06	9	4.59E+03	9.57E+02	4
EPB41L2	1.04E+05	1.42E+05	2			0
DNM1L	5.31E+05	7.56E+05	3	3.36E+03		1
TTN	1.31E+06	2.88E+06	7	9.61E+03	7.26E+03	5
CCT8	2.14E+05	1.25E+05	3			0
SERPINB1	6.71E+04	5.90E+04	8			0
GANAB; HEL-S-164nA	3.89E+05	8.68E+05	10			0
TUBA3E	1.51E+05	1.54E+05	6	1.97E+04	2.70E+04	5
NDRG2	4.69E+05	3.74E+05	4	4.05E+03	2.21E+03	3
TPD52	8.66E+04	1.02E+05	6	1.84E+04		1
FAH	1.35E+05	1.43E+05	11	2.45E+04	1.99E+04	5
PKLR	1.29E+05	1.49E+05	7			0
GH1	1.56E+05	1.15E+05	3			0
NFASC	1.65E+05	2.08E+05	5	5.60E+04	9.52E+04	6
DYNC1I2	2.85E+05		1			0
SYNJ1	8.55E+04	4.54E+04	4	2.00E+04	8.81E+03	2
SEPTIN2	6.10E+04	3.61E+04	9			0
SNX2	7.67E+04	5.60E+04	9	2.80E+03	3.71E+02	2
FGFR1 (Iso 21)	8.01E+04	2.91E+04	2	1.98E+04	1.72E+04	3
CGREF1	8.02E+04	4.60E+04	4	3.82E+03		1
NPEPPSL1	7.88E+04	8.18E+04	4			0
FHL1	5.74E+04	2.11E+04	5	5.91E+03	6.05E+02	2
RDX	1.65E+05	2.04E+05	9	2.17E+03		1
BTD	3.44E+04	2.18E+04	5			0
EIF5A2	1.09E+05	1.51E+05	4	5.89E+03	4.44E+03	5
TPD52	3.02E+04	1.25E+04	2			0
UGDH	1.28E+05		1			0
FUBP1	3.92E+04	1.66E+04	2	8.60E+04	1.22E+05	5
SLK	7.08E+04	6.16E+04	6			0
LRBA	1.00E+05	1.89E+04	2	5.15E+03	3.00E+02	2
AIFM1	7.21E+04	6.00E+04	6	3.33E+03		1
HUWE1	3.51E+04	2.52E+04	3			0
HNRNPA1; HNRPA1	5.01E+04	1.34E+04	3			0
AKAP9	8.33E+04	8.64E+04	7	6.24E+03	3.09E+03	4
AP2A2	6.52E+04	6.84E+04	2	2.89E+03		1
LDHA	5.12E+04	4.36E+04	6	6.56E+03	3.83E+03	2
DPP3	1.34E+05		1			0
ITIH1	2.83E+05		1	5.18E+03	1.89E+03	2
AHCYL2	4.68E+04	2.94E+04	6			0
VCAM1	1.84E+05	2.16E+05	2	1.08E+04		1
NSFL1C	6.64E+04	1.06E+04	3			0
NRCAM	5.15E+04	4.78E+04	8	1.71E+03		1
NDRG2	7.53E+04	5.61E+04	3			0
TPI1	1.60E+04	1.15E+04	2			0
RANBP1	1.63E+04	3.76E+03	4			0
ATP1A1	1.44E+04		1	3.09E+03		1
PLTP			0	5.59E+03		1
